# The Dawn of Open Access to Phylogenetic Data

**DOI:** 10.1371/journal.pone.0110268

**Published:** 2014-10-24

**Authors:** Andrew F. Magee, Michael R. May, Brian R. Moore

**Affiliations:** Department of Evolution and Ecology, University of California Davis, Davis, CA, United States of America; Texas A&M University, United States of America

## Abstract

The scientific enterprise depends critically on the preservation of and open access to published data. This basic tenet applies acutely to phylogenies (estimates of evolutionary relationships among species). Increasingly, phylogenies are estimated from increasingly large, genome-scale datasets using increasingly complex statistical methods that require increasing levels of expertise and computational investment. Moreover, the resulting phylogenetic data provide an explicit historical perspective that critically informs research in a vast and growing number of scientific disciplines. One such use is the study of changes in rates of lineage diversification (speciation – extinction) through time. As part of a meta-analysis in this area, we sought to collect phylogenetic data (comprising nucleotide sequence alignment and tree files) from 217 studies published in 46 journals over a 13-year period. We document our attempts to procure those data (from online archives and by direct request to corresponding authors), and report results of analyses (using Bayesian logistic regression) to assess the impact of various factors on the success of our efforts. Overall, complete phylogenetic data for 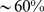 of these studies are effectively lost to science. Our study indicates that phylogenetic data are more likely to be deposited in online archives and/or shared upon request when: (1) the publishing journal has a strong data-sharing policy; (2) the publishing journal has a higher impact factor, and; (3) the data are requested from faculty rather than students. Importantly, our survey spans recent policy initiatives and infrastructural changes; our analyses indicate that the positive impact of these community initiatives has been both dramatic and immediate. Although the results of our study indicate that the situation is dire, our findings also reveal tremendous recent progress in the sharing and preservation of phylogenetic data.

## Introduction

Archiving and sharing published data is a social contract that is integral to the scientific enterprise [1]. Sharing published data advances the scientific process by: (1) exposing published results to independent verification (to identify errors and discourage fraud); (2) providing the pedagogical material for educating students and training future researchers; (3) acting as a test bed to guide the development of new methods, and; (4) providing a basis to identify and pursue new questions via synthesis/meta-analysis [2]. Additionally, archiving published data protects our scientific investment, avoiding needless costs of data regeneration in terms of time, money, and environmental impact [3].

These considerations are particularly germane to phylogenetic data, which include both alignments (estimates of the positional homology of molecular sequences) and phylogenetic trees (estimates of the evolutionary relationships among species). Phylogenetic trees for individual groups are inherently synthetic—combination of these ‘twigs’ provides a natural approach for elucidating the entire Tree of Life, *c.f.*, [4], [5]. Additionally, phylogenetic data have tremendous potential for reuse, often in ways that were completely unanticipated by the original studies: because they provide an explicit evolutionary perspective, phylogenies have become central to virtually all areas of research in evolutionary biology, ecology, molecular biology and epidemiology [6], [7], [8]. Moreover, the generation of phylogenetic data is an increasingly arduous and technical enterprise. Clearly, phylogenetic data are a precious scientific resource that must be preserved and shared in order to realize their full potential.

The vast majority of phylogenies are estimated from molecular (primarily nucleotide) sequence data. Although GenBank and similar public archives provide a robust (albeit imperfect, [9]) backstop against the complete loss of the *raw* sequence data, these databases do not safeguard the associated *phylogenetic* data: the alignments estimated from raw sequence data, and the trees inferred from those alignments. Multiple sequence alignment—the process of estimating the positional homology of each nucleotide site comprising DNA sequences—is a difficult inference problem for which many approaches have been proposed [10], [11]. Different algorithms (or different settings for a given algorithm) may yield dramatically different estimates of the alignment that, in turn, can substantially impact estimates of phylogeny [12], [13]. Moreover, the majority of phylogenetic studies are based on alignments that are subjected to ‘manual adjustment’ after being estimated using formal methods [14], which effectively destroys the possibility of replicating published alignments from the corresponding raw sequence data. Even if the alignment could be dependably reproduced, replicating the published phylogeny requires a precise description of how the phylogenetic analysis was performed, details that are typically not provided in phylogenetic studies [15]. Finally, even if the alignment and details of the analysis were available, re-generating the phylogeny remains a non-trivial proposition: the analysis of a single dataset may require hundreds or thousands of compute hours [16].

These issues have been appreciated for some time [17], and motivated the development of a specialized online archive for phylogenetic data, TreeBASE [18], more than 20 years ago. Despite such noble efforts, it is increasingly evident that the loss of phylogenetic data is catastrophic: recent surveys estimate that 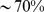 of published phylogenetic data are lost forever [8], [19], [20]. In response to this crisis, several recent community initiatives have been proposed to encourage the preservation and sharing of phylogenetic data. These include policy initiatives both by funding agencies (the NSF Data Management Plan established in 2011 that requires the preservation of data generated by funded research), and by journals/publishers (the establishment of the Joint Data Archiving Policy, JDAP, by a consortium of prominent journals requiring the submission of data to online archives as a condition of publication [21], [22], [23], [24], [25]), and the establishment of a new online archive for evolutionary and ecological data, Dryad [26].

We set out to perform a meta-analysis exploring the empirical prevalence of temporal changes in rates of lineage diversification. To this end, we sought to collect the phylogenetic data from studies using the two most common statistical phylogenetic approaches for detecting temporal shifts in diversification rate; *i.e.*, the ‘gamma’ statistic (‘method 1’ [27]) and the ‘birth-death likelihood’ (‘method 2’ [28]) methods. To be included in our meta-analysis, we required two key data files from each published empirical study: (1) an alignment of nucleotide sequence data, and (2) an ultrametric tree (where the branch lengths are rendered proportional to relative or absolute time). We document our attempts to procure these data (both via searches of online archives and by direct solicitation from the corresponding authors), and describe results of analyses exploring various factors associated with the availability of phylogenetic data. We assess a number of correlates—the age of the study, the impact factor and data-sharing policy of the publishing journal, the status of the solicitor, etc.—with a focus on revealing the efficacy of recent community initiatives to ensure the preservation and promote the sharing of published phylogenetic data.

## Methods

In this section, we document our attempts to procure phylogenetic data from a large and random sample of studies exploring temporal variation in rates of lineage diversification published over a 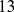-year period. We first describe how we sought to collect these data, and then describe the analyses we performed to gauge the success of our efforts.

### Data Collection

During the months of August and September, 2013, we searched for articles citing the two methods papers using the the Google Scholar cited-reference search tool. Our search identified a total of 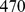 citing articles (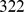 and 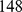 for methods 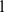 and 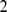, respectively). Of these, 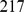 articles involved empirical analyses (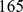 and 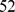 using methods 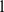 and 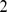, respectively).

For each study, we captured bibliometric data on authorship, publication month and year, and the name and impact factor of the publishing journal. We also recorded the data-sharing policy of the publishing journal and whether it was a member of the JDAP initiative at the time of publication. Specifically, we ascertained the data-sharing policy for each of the 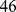 journals from the corresponding ‘instructions to authors’ documentation (see *Journal Policies* section of File S1). Following [29], we categorized journals that made *no mention* of data sharing as having *no policy*; those that *encouraged* authors to share data upon publication were scored as having a *weak policy*; those that *required* data sharing as a condition of publication were scored as having a *strong policy*; and those that were members of the JDAP initiative were scored as having *JDAP membership*. Finally, we noted whether the studies acknowledged funding support from the National Science Foundation (NSF).

**Figure 1:**
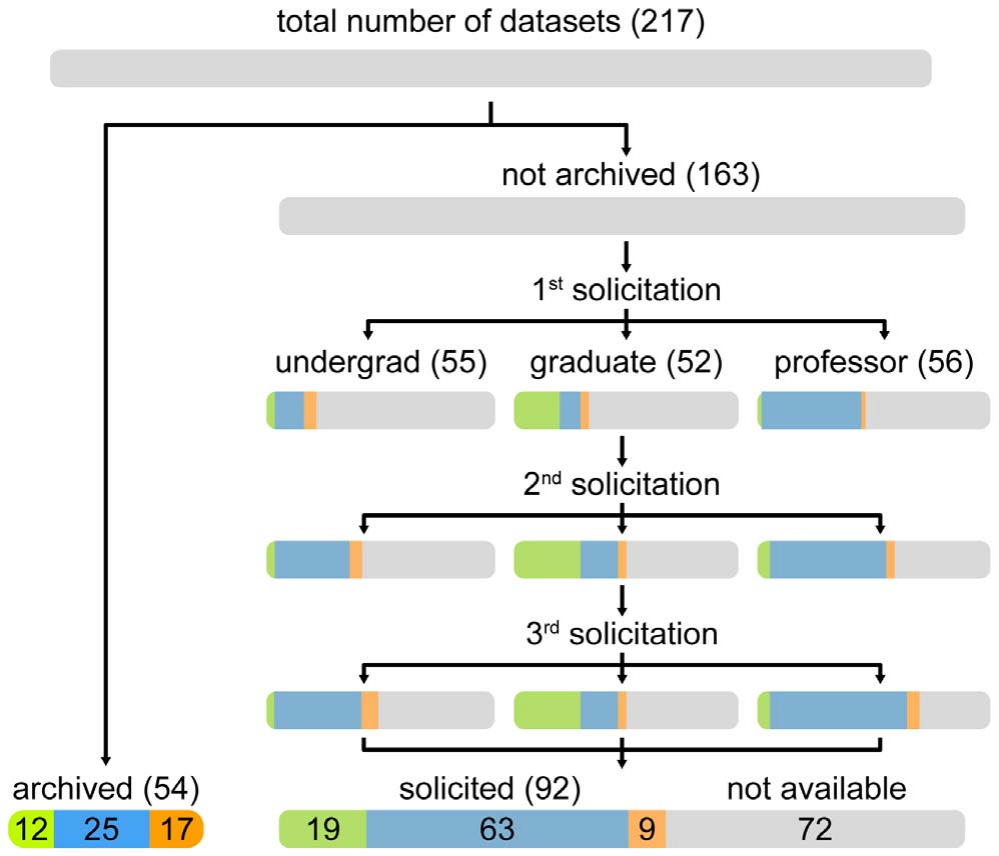
Flowchart of data acquisition. We identified a total of 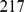 articles exploring temporal variation in rates of lineage diversification. Data for 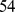 of these studies were archived in online repositories; data for the remaining 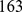 studies were solicited by direct requests to the corresponding author by an undergraduate student (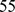 studies), a graduate student (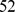), or a professor (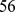). A maximum of three requests were made at weekly intervals. Recovered phylogenetic data comprised tree files (green), alignment files (orange), or both (blue). Datasets not obtained after the third request were deemed unavailable (gray).

For each study, we assessed whether data were available online by first searching each article for various keywords (“Dryad”, “TreeBASE”, etc.), and pursued any links or references to archived data. If data could not be sourced directly from the article itself, we proceeded to examine any associated Supplemental Material files using a similar strategy. Articles that did not submit their data to online repositories were targeted for direct solicitation using a semi-automated, multi-step approach (Figure 1). Specifically, we wrote ‘templates’ for three sequential messages comprising an initial, a followup, and a final request for published phylogenetic data (see *Example Template Messages* section of File S1). In the messages, we identified ourselves, provided details of the requested data, and explained the reason for our request; that is, we explained that we were gathering data for a meta-analysis evaluating the prevalence of temporal changes in diversification rate, and we sought the sequence alignment and ultrametric tree files that were the used to assess temporal changes in diversification rates in the published study.

Each of the three message templates contained ‘fields’ for several variables, including: the name and status of the solicitor; the name and email address of the corresponding author; and the year and title of the published article. We divided the solicitations evenly (and randomly) between the three of us. This was intended both to share the burden equably, and also to assess any effect of the solicitor status, which comprised a professor (BRM), a graduate student (MRM) and an undergraduate student (AFM). We then generated messages using R scripts that populated the fields of the templates with the relevant information from the spreadsheet (we provide the message templates and R scripts in File S1). Messages were sent at weekly intervals. If we received a response, the corresponding author was precluded from receiving subsequent generic email messages, and we corresponded with them on an individual basis. We recorded various details of each response, including whether the recipient sent the requested alignment file and/or tree file. Datasets not obtained at the end of this process were deemed unavailable.

We assembled a data table summarizing the information gathered for the 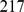 studies (see File S2). Following [30], the data table has been anonymized to protect the identity of corresponding authors (*i.e.*, with regard to who did or did not archive and/or share phylogenetic data from published studies). However, a key is available upon request to allow details of our analyses to be independently verified. In any case, the issues that we document are general and should not be use to impugn the academic integrity of the individual researchers.

### Data Analysis

We used Bayesian logistic regression to explore correlations between data availability and several variables. Under this approach, a *trial* is an attempt to recover data for a particular study either from online archives or by direct solicitation, which we deem a *success* if we received data for that study. The outcomes of a set of 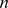 trials are contained in a data vector 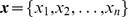, where 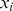 is 1 if we obtained the relevant data for study 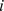 and is 0 otherwise. The outcome of each trial depends on a set of 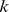 *predictor variables* that may be continuous (*e.g.*, the journal impact factor) or discrete (*e.g.*, the status of the solicitor). An 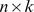 matrix 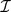, the *design matrix*, describes the relationships between trials and predictor variables: 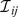 is the value for predictor variable 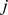 for trial 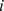. *Parameters* relate the values of each predictor variable to the probability of success of each trial, and are described by the parameter vector 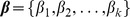, where 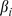 is the contribution of parameter 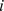 to the probability of success.

In a Bayesian framework, we are interested in estimating the joint posterior probability distribution of the model parameters 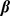 conditional on the data 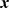. According to Bayes' theorem,
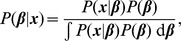
the *posterior probability* of the model parameters, 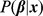, is equal to *likelihood* of the data given the model parameters, 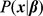, multiplied by the *prior probability* of the parameters, 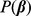, divided by the *marginal likelihood* of the data.

Given the design matrix 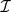, the outcomes of each of the 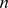 trials are conditionally independent, so that the likelihood of 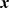 is the product of the likelihoods for each individual trial:
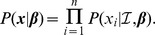



The likelihood of observing the outcome of a particular trial is
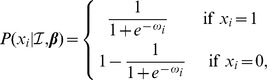
where



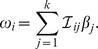



We specified a multivariate normal prior probability distribution on the 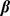 parameters with means 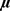 and covariance matrix 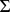. The complexity of the marginal likelihood precludes an analytical solution to the posterior probability distribution. Accordingly, we approximated the posterior probability distribution using the Markov chain Monte Carlo algorithm implemented in the R package BayesLogit [31], [32]. This program uses conjugate prior and posterior probability distributions (via Polya-Gamma-distributed latent variables), which permits use of an efficient Gibbs sampling algorithm to approximate the joint posterior distribution of 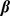 conditional on the data.

We defined a set of predictor variables based on the bibliometric metadata captured for each study. We included an *intercept* predictor variable to describe the background probability of procuring data. We treated *age* (*i.e.*, months since publication) and *journal impact factor* as continuous predictor variables, and *journal policy*, *NSF funding*, and *solicitor status* as discrete predictor variables. Discrete predictor variables for logistic regression are generally binary, assuming values of 0 or 1. A few of our discrete bibliometric metadata, however, had more than two possible categories. We therefore adopted an *indicator-variable* approach in which predictor variables with 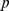 categories are discretized into 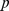 distinct indicators; each study in a particular predictor category was then assigned a 1 for the corresponding indicator variable. Under this approach, studies published in journals with no data-sharing policy were assigned a 1 for the *no policy* variable, studies published in journals with a strong policy were assigned a 1 for the *strong policy* variable, and studies published in journals that were members of the JDAP initiative at the time of publication were assigned a 1 for the *JDAP membership* variable. For the studies included in our direct-solicitation campaign, we also assigned values for solicitor status: datasets solicited by an undergraduate student were scored as 1 for the *undergraduate student* variable, while those solicited by a professor were scored as 1 for the *professor* variable. In order to avoid overparameteriziation of the logistic model, we did not assign indicator variables for the *weak-policy* or *graduate-student* variables. Accordingly, the values for *no policy*, *strong policy*, and *JDAP membership* parameters are interpreted as effects relative to weak policies; similarly, the values for *undergraduate student* and *professor* parameters are interpreted as effects relative to a graduate student. Details of the predictor variables and interpretations of the corresponding parameters are summarized in Table 1. We tested whether our predictor variables were correlated (by calculating *variance inflation factors*, [33]), since this can influence interpretations of parameter estimates; however, correlations among our predictor variables appear to be minimal (see Figure S1 and Table S2 in the *Multicollinearity Analysis* section File S1).

**Table 1:** Summary of logistic model parameters and their interpretation.

Parameter	Predictor variable	Interpretation
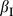	*intercept*	The “base” log-odds of retrieving the data, irrespective. of other model parameters.
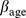	*age*	The change in log-odds of retrieving the data per month of the study's age.
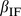	*impact factor*	The change in log-odds of retrieving the data per unit impact factor of the journal in which the study was published.
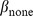	*no policy*	The change in log-odds of retrieving the data if the study was published in a journal with no data-availability policy (relative to a weak policy).
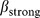	*strong policy*	The change in log-odds of retrieving the data if the study was published in a journal with a strong data-availability policy (relative to a weak policy).
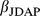	*JDAP membership*	The change in log-odds of retrieving the data if the study was published in a member of the JDAP initiative beginning 2011 (relative to a weak policy).
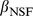	*NSF funding*	The change in log-odds of retrieving the data if the study reported NSF funding beginning 2011.
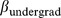	*undergraduate student*	The change in log-odds of retrieving the data if it was solicited by an undergraduate student (relative to a graduate student).
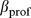	*professor*	The change in log-odds of retrieving the data if it was solicited by a professor (relative to a graduate student).
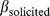	*solicited*	The change in log-odds of retrieving the data if it was solicited (relative to archived).

We analyzed various subsets of our data table in order to understand the relative importance of the predictor variables on different aspects of data availability. Specifically, we defined subsets of our data table based on whether study data were sought: (1) by queries to online archives, (2) by direct solicitation from the corresponding author, or (3) either by queries to online archives *or* by direct solicitation. We further parsed our data table based on whether we successfully procured: (1) *only* trees (*i.e.*, the trial outcome was 1 if we acquired a tree and no alignment, and 0 otherwise); (2) *only* alignments; (3) either alignments *or* trees (*i.e.*, the trial outcome was 0 if we acquired no data, and 1 otherwise), and; (4) both alignments *and* trees (*i.e.*, the trial outcome was 1 if we acquired both an alignment and a tree). This defined 16 (overlapping) subsets of our data table. Note that not all predictor variables apply to every subset of our data table; *e.g.*, the solicitor-status variable, *undergraduate*, only applies to data that were directly solicited. Details of the data subsets and their predictor variables are summarized in Table S1.

We estimated parameters for each data subset by performing four independent MCMC simulations, running each chain for 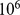 cycles and saving every 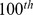 sample to reduce autocorrelation and file size. We assessed the performance of all MCMC simulations using the Tracer [34] and coda [35] packages. We monitored convergence of each chain to the stationary distribution by plotting the time series and calculating the Geweke diagnostic (*GD* [36]) for every parameter. We assessed the mixing of each chain over the stationary distribution by calculating both the potential scale reduction factor (*PSRF* [37]) diagnostic and the effective sample size (*ESS* [38]) for all parameters. Values of all diagnostics for all parameters in all MCMC simulations indicate reliable approximation of the stationary (joint posterior probability) distributions: *e.g.*, 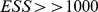; 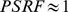; *GD* 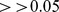 (Tables S3
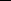
S14 in File S1). Additionally, we assessed convergence by comparing the four independent estimates of the marginal posterior probability density for each parameter, ensuring that all parameter estimates were effectively identical and SAE compliant [38]. Based on these diagnostic analyses, we discarded the first 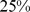 of samples from each chain as burn-in, and based parameter estimates on the combined stationary samples from each of the four independent chains (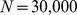). We assessed the sensitivity of our estimates to the chosen priors by computing the Kullback-Leibler divergence [39] between the marginal posterior probability density and the corresponding prior probability density for each parameter. The KL divergence was large for all marginal posterior probability densities (indicating limited impact of the prior on parameter estimates), with the notable exception of the *JDAP* parameter for solicited data (see Figures S2–S3 in the *Prior Sensitivity Analysis* section in File S1). The low KL divergence of the *JDAP* parameter for solicited studies reflects the limited information available for estimating this parameter: we directly solicited only 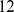 datasets from studies published in JDAP journals.

## Results and Discussion

**Figure 2:**
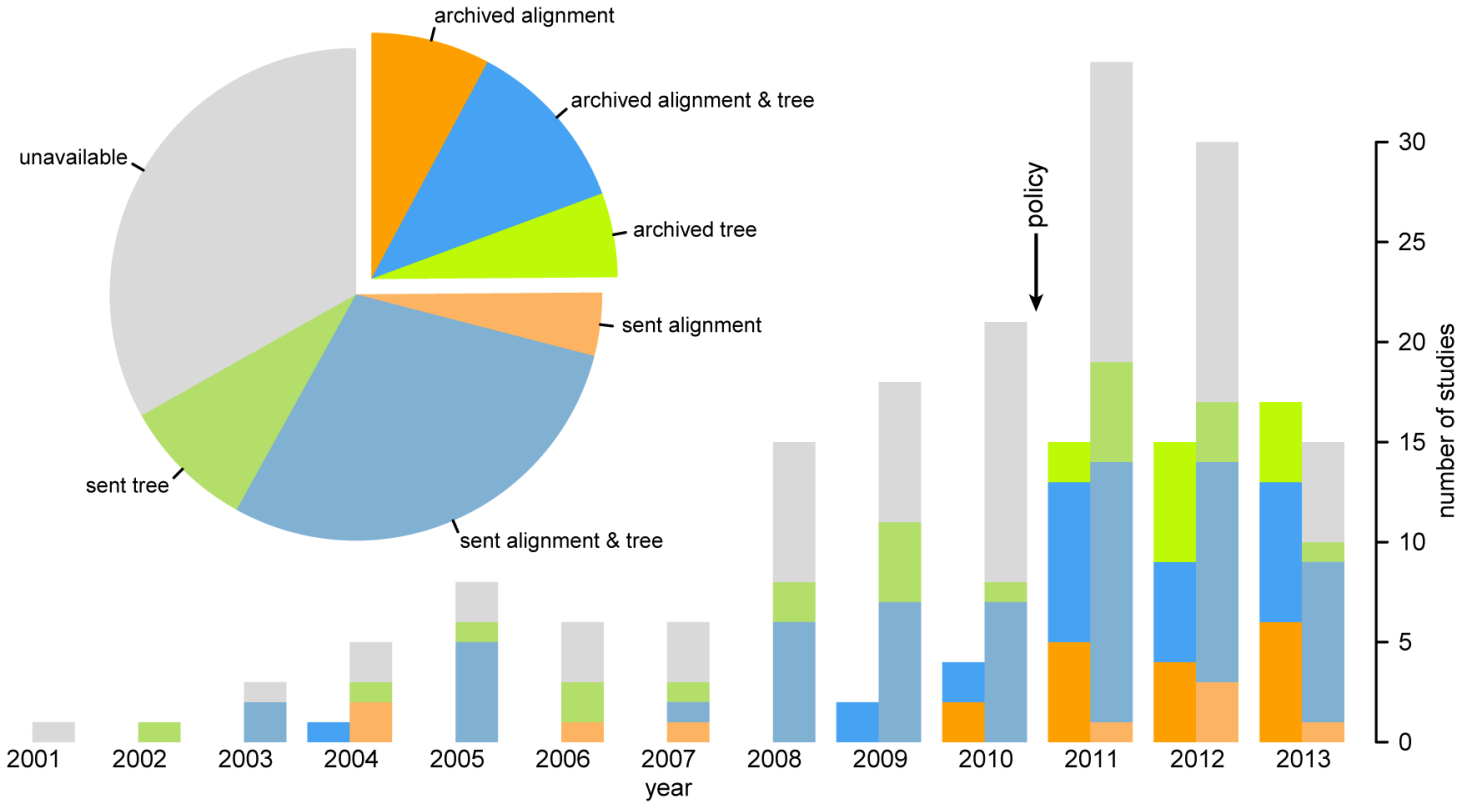
Detailed breakdown of data availability. The number of studies with available phylogenetic data—as tree files (green), alignments files (orange) or both (blue), procured either from online archives or by direct request—organized by year of publication (barplot). Phylogenetic data of some kind (tree and/or alignment files) were available from an online archive for approximately 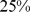 of the studies, and additional data were successfully solicited by direct request for 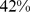 of the studies. Complete datasets were unavailable for 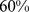 of published studies, and data of any kind were unavailable for 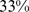 of studies (gray). The ‘policy’ arrow indicates the onset of several community initiatives to improve the sharing and preservation of evolutionary (including phylogenetic) data, which coincides with a marked increase in the deposition of phylogenetic data to online archives. For each pair of barplots, the left/right bars correspond to archived/solicited data, respectively. Grayscale image available at http://dx.doi.org/10.6084/m9.figshare.1148872.

Overall, our efforts secured complete phylogenetic data for 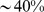 of the published studies (Figure 2). Accordingly, invaluable phylogenetic data for more than half of these studies are effectively lost to science. From online archives, we successfully procured *complete* phylogenetic data (both the tree and alignment files) for 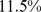 of the studies, and *partial* datasets (either the tree or alignment files) for an additional 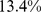 of the studies were archived: 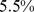 of these cases had only tree files, 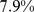 had only alignment files. Of these online accessions, 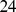 were archived in Dryad, 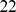 in TreeBASE, and 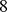 as supplemental files on journal websites. Our (in)ability to recover phylogenetic datasets from online archives over the *entire* 13-year period is comparable to that of recent reports regarding phylogenetic data—where archival rates range from 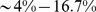 [8], [19], [40]—and also falls within the scope of archival rates for non-phylogenetic data, which range from 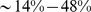 [41], [42], [43]. However, our results also reveal a dramatic increase in the archiving of phylogenetic data since 2011; *e.g.*, datasets from more than half of the studies published in 2013 were deposited in online archives (Figure 2).

Our direct-solicitation campaign entailed the exchange of 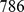 emails over the course of four weeks (BRM: 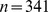; MRM: 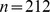; AFM: 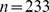). We received responses to 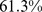 of the 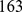 messages we sent to corresponding authors (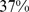, 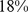, and 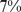 after the first, second and third message, respectively), 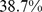 of the authors never responded to any messages (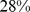, 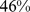, and 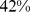 for BRM, MRM, and AFM, respectively). Although 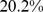 of the messages were initially undeliverable (owing to invalid/obsolete email addresses), we were able to resolve contact information for all but 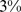 of the corresponding authors (by performing Internet searches and/or contacting study co-authors). Our 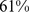 response rate is comparable to that of previous studies. A recent survey [19] reported a 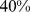 response rate to direct requests for phylogenetic data, which falls within the range for studies involving non-phylogenetic data: *e.g.*, 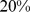 for medical/clinical trial data [44]; 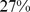 for psychological trial data [45]; and 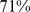 for population-genetic data [43].

By directly contacting corresponding authors, we successfully procured complete phylogenetic datasets for 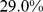 of the published studies, and partial datasets for an additional 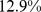 of the studies: 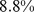 of corresponding authors sent only tree files, and 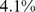 sent only alignment files (Figure 2). Our success in procuring complete 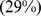 or some form 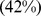 of phylogenetic data by direct solicitation compares favorably to the 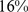 recovery rate of a recent study [19], but again is within the range reported for non-phylogenetic data; *e.g.*, 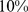 for medical/clinical trial data [44]; 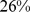 for psychological-trial data [45]; 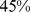 for gene-expression data [46]; 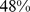 for cancer microarray data [41]; 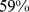 for population-genetic data [43].

**Figure 3:**
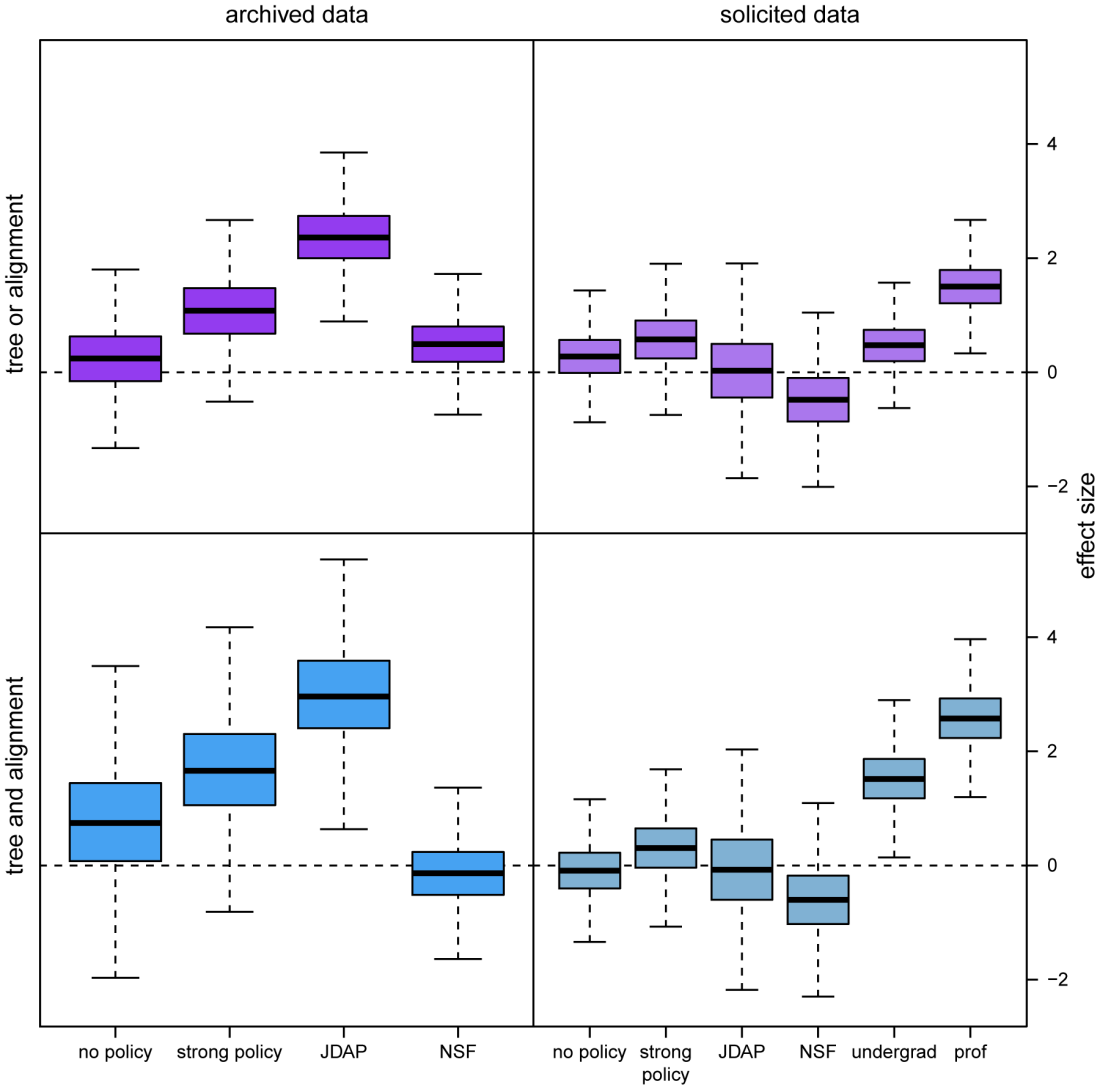
Correlates of data availability. We used Bayesian logistic regression to estimate the effect of several variables on the on the probability that phylogenetic datasets were either available from a public archive (left column) or could be successfully procured by direct solicitation (right column). Specifically, for all datasets we explored the effect of the data-sharing policy of the publishing journal (scored as *none*, *weak*, *strong*, or *JDAP membership*) and the impact of funding-agency policy (*NSF*). For solicited datasets, we also assessed the impact of solicitor status (*undergraduate*, *graduate*, or *professor*). We estimated effects of these variables on our ability to successfully procure *either* the tree or alignment files (top panels), or *both* the tree and alignment files (bottom panels) for a given study. The estimated effect size for a given variable reflects its contribution to the probability of successfully acquiring the data. For each variable, the marginal distribution of its estimated effect size is summarized as a boxplot, indicating the median effect (solid line), 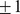 interquartile range (box), and 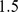 interquartile range (whisker) of the corresponding posterior probability distribution. Journal-policy effects are relative to the effect of a weak policy, and solicitor-status effects are relative to that of graduate student. The predictor variables and interpretation of the corresponding parameters are described in Table 1.

The results of our logistic-regression analysis provide insights into factors associated with the availability of published phylogenetic data (Figure 3; Tables 2–3). Studies published in journals with strong data-sharing policies are more likely to archive both complete (tree and alignment files) and incomplete (tree or alignment files) phylogenetic data, and are also more likely to provide complete and incomplete phylogenetic data upon direct request. Strikingly, the availability of phylogenetic data (via online archives or direct solicitation) from studies published in journals with weak data-sharing policies is comparable to (or slightly worse) than that of studies published in journals with no data-sharing policy, *c.f.*, [29], [43]. This observation substantiates recent calls for establishing strong (and stringently enforced) data-sharing policies [2], [19], [20], [29], [44]. The efficacy of such policies is evident for studies published in JDAP journals. Surprisingly, there is a *low* probability of directly soliciting data for studies published in JDAP journals. However, this likely reflects the fact that the data from these studies are so often available in online archives that there is essentially no *need* for direct solicitation; indeed, datasets were only solicited from 12 studies published in JDAP journals (*c.f.*, Figure S3).

**Table 2:** Relative probability of obtaining phylogenetic data from online archives.

	alignments or trees			alignments and trees		
		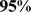 HPD			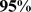 HPD	
	mean	lower	upper	mean	lower	upper
*no policy*	1.17	0.42	2.35	1.87	0.05	10.95
*strong policy*	1.83	0.79	3.52	3.92	0.32	21.84
*JDAP membership*	2.76	1.40	5.46	8.58	1.86	54.19
*NSF funding*	1.37	0.67	2.33	0.91	0.20	2.09

**Table 3:** Relative probability of procuring phylogenetic data by solicitation.

	alignments or trees			alignments and trees		
		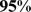 HPD			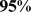 HPD	
	mean	lower	upper	mean	lower	upper
*no policy*	1.16	0.66	1.78	0.94	0.35	1.82
*strong policy*	1.32	0.78	2.08	1.31	0.44	2.49
*JDAP membership*	1.03	0.30	1.85	1.03	0.09	2.52
*NSF funding*	0.76	0.27	1.34	0.65	0.12	1.40
*undergraduate student*	1.27	0.79	1.93	2.76	1.16	6.10
*professor*	1.78	1.19	2.82	4.21	1.80	9.57

Our analyses also indicate that corresponding authors are more likely to grant data requests from faculty than from students (Figure 3). This may simply reflect the fact that the faculty solicitor (BRM) is acquainted with a larger proportion of the corresponding authors. However, this does not explain why corresponding authors are more likely to provide data to undergraduate than to graduate students. An alternative (but not mutually exclusive) explanation involves the perceived risks of data sharing. Authors may be reluctant to share published data for fear (reasonable or not) that reanalysis may identify errors and/or reach contradictory conclusions [47], [48]. This idea has, in fact, been substantiated by a recent study demonstrating that reluctance to share published data is significantly correlated with weaker evidence and a higher prevalence of apparent errors in the reporting of statistical results [30]. Accordingly, corresponding authors may perceive requests from undergraduate students to present less potential risk than those from graduate students, whereas the potential risks presented by faculty requests are balanced by their greater familiarity to the authors.

**Figure 4:**
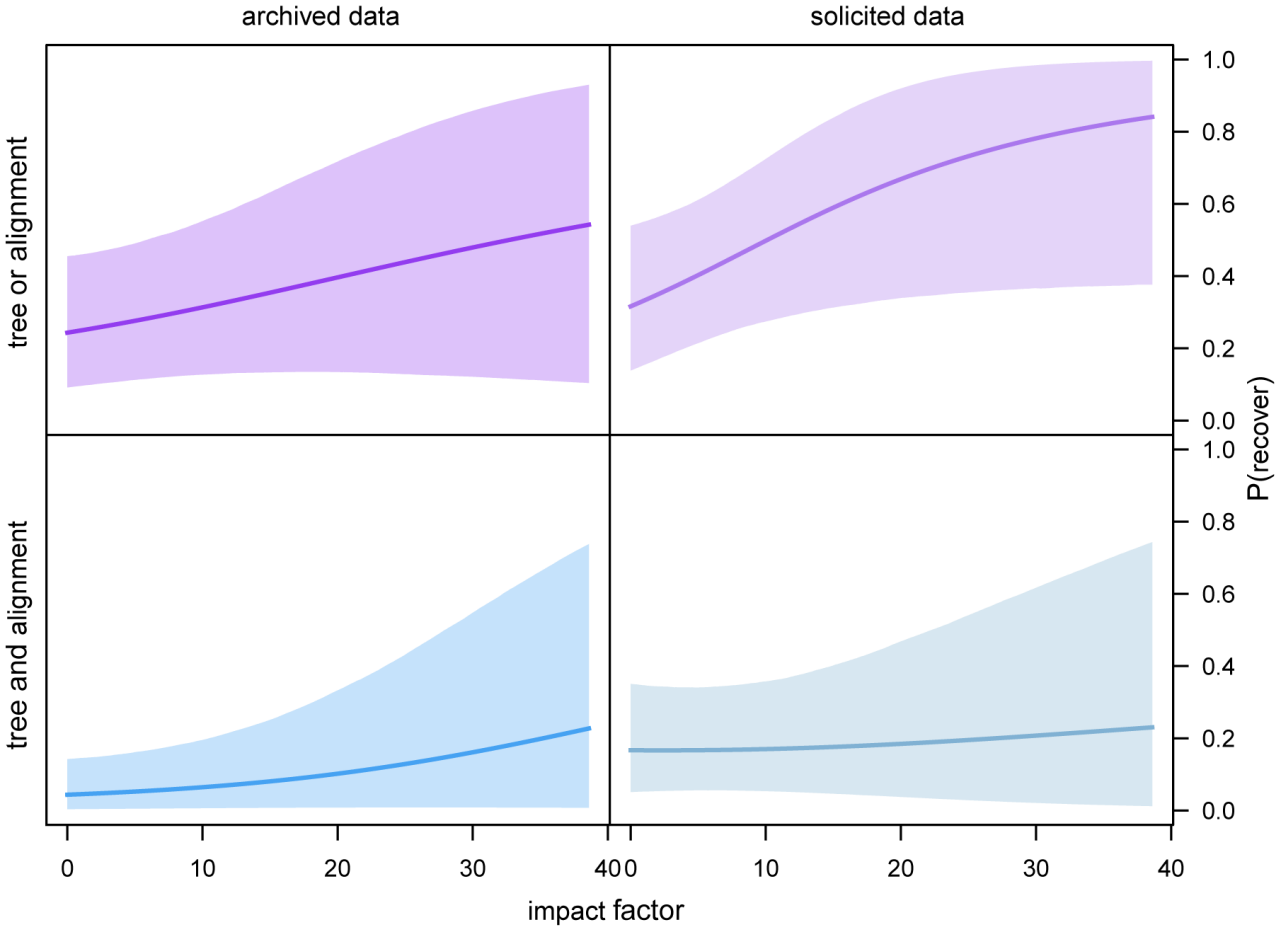
Availability of phylogenetic data as a function of impact factor. We estimated the effect of the impact factor of the publishing journal on our ability to procure partial (top panels) and complete (bottom panels) phylogenetic datasets from online archives (left panels) or by direct solicitation (right panels). Generally, studies published in journals with a higher impact factor are more likely to both deposit the corresponding (partial or complete) datasets in online archives and to provide those data upon direct request. The shaded areas reflect the 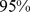 credible intervals of the estimates.

The influence of journal impact factor on data availability might also be interpreted from the perspective of perceived risk. As for non-phylogenetic data [29], [43], our analyses indicate that studies published in journals with a higher impact factor are more likely to both deposit their phylogenetic data in online archives and provide these data upon direct request (Figure 4). If willingness to share published data is correlated with the quality of the research [30], and if research quality is correlated with the impact factor of the publishing journal, then journal impact factor should positively predict data availability. An alternative (perhaps less conspiratorial) explanation for the correlation between journal impact factor and data availability invokes an indirect effect of journal impact factor on journal data-sharing policy. That is, by virtue of their greater prestige, journals with higher impact factors may have greater reign to impose stronger (and more strictly enforced) data-sharing policies on contributing authors [43].

**Figure 5:**
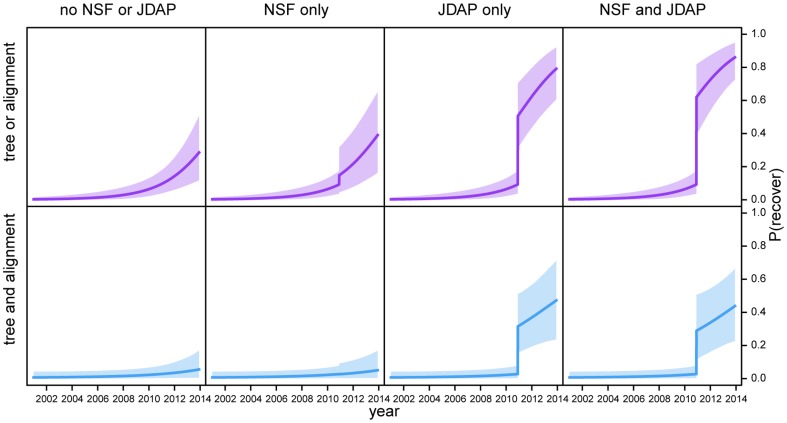
Availability of archived phylogenetic data as a function of age. We estimated the effect of publication age on our ability to procure partial (top panels) and complete (bottom panels) phylogenetic datasets from online archives. Overall, the probability of recovering archived phylogenetic data increases toward the present, with a conspicuous recent increase for partial datasets (left panels). The recent surge of archived phylogenetic data likely reflects recent policy changes (middle panels): studies with NSF funding are more likely to archive alignment (but not tree) files (*c.f.*, Table S15); whereas studies published in journals with JDAP membership are dramatically more likely to archive both partial and complete phylogenetic datasets. The effects of these policy initiatives are not strictly additive (right panels): the correlation of these predictor variables suggests that studies published in JDAP journals are likely to have NSF funding. Shaded areas reflect the 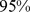 credible intervals.

**Figure 6:**
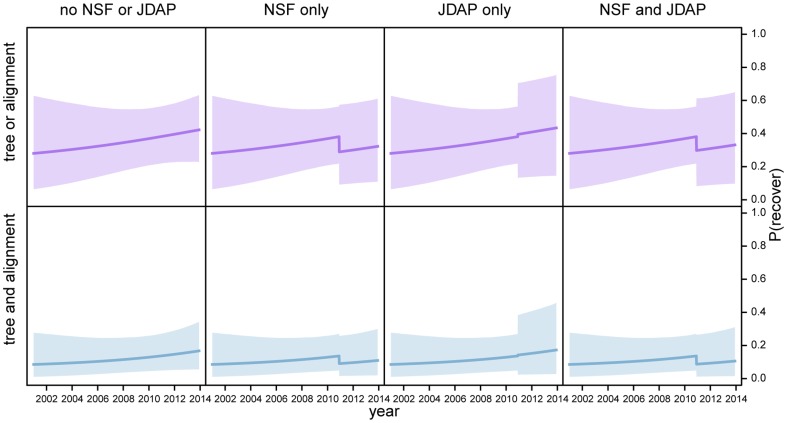
Availability of solicited phylogenetic data as a function of age. We estimated the effect of publication age on our ability to procure partial (top panels) and complete (bottom panels) phylogenetic datasets by direct solicitation. Overall, the probability of successfully recovering phylogenetic data decreases over time (left panel). Paradoxically, the probability of soliciting data from studies with NSF funding and/or published in JDAP journals is *lower* than that for studies without NSF funding and/or published in non-JDAP journals. However, this likely reflects the fact that the data from these studies are so often available in online archives that there is essentially no *need* for direct solicitation. Shaded areas reflect the 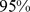 credible intervals.

As in previous studies [49], [50], our results indicate that data availability decreases markedly over time. Several corresponding authors reported that the requested datasets had been misplaced or had been lost due to hard-drive failures. As noted above, there appears to be a distinct uptick in the availability of data from studies published since 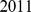; this trend was particularly pronounced for archived data (Figure 5). This pattern may simply indicate that the decay of archived phylogenetic data is nonlinear. Our findings, however, indicate that the recent surge in archived phylogenetic data is attributable to policy changes. Studies with NSF funding are 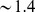 times more likely to archive some kind of phylogenetic data (tree or alignment files), but are actually *less* likely to archive complete phylogenetic data (Table 2). Curiously, the NSF mandate has led to a drastic increase in archiving alignment (but not tree) files (Table S15; see also Tables S16–S17 in File S1). By contrast, studies published in journals with JDAP membership are 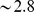 and 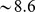 times more likely to archive partial and complete phylogenetic datasets, respectively (Table 2; Figure 5). Paradoxically, the probability of successfully soliciting data from studies with NSF funding and/or published in JDAP journals is *lower* than that for studies without NSF funding and/or published in non-JDAP journals (Figure 6). However, this likely reflects the decreased demand for these data by direct solicitation.

## Summary

Phylogenetic data are a precious scientific resource: molecular sequence alignments and phylogenies are expensive to generate, difficult to replicate, and have seemingly infinite potential for synthesis and reuse. At face value, our results support the conclusion of recent studies [8], [19], [20] that the loss of phylogenetic data is catastrophic: complete phylogenetic datasets have been lost for 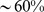 of the studies we surveyed. Our results also identify factors associated with (phylogenetic) data availability that have been implicated by previous studies: the probability of procuring phylogenetic data is strongly predicted the age of the study, and the data-sharing policy and impact factor of the publishing journal.

Unlike previous studies, however, our survey of phylogenetic datasets spans important policy initiatives and infrastructural changes, and so provides an opportunity to assess the efficacy of those recent measures. Overall, the positive impact of these community initiatives has been both substantial and immediate. Even at this very early stage—spanning the first three years since the introduction of these policies—the archival rate of phylogenetic data has increased dramatically. Specifically, the proportion of studies that archived partial or complete phylogenetic data since 2011 has increased 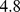-fold and 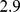-fold, respectively. Moreover the proportion of archived phylogenetic data has increased each year since the policy changes, and deposition rates of phylogenetic data to Dryad have been 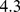 times that of the more established TreeBASE archive. The prospects for future progress along these lines appear promising: membership of the JDAP consortium has almost tripled in the three years since its formation.

Although recent policy initiatives have had a clear and welcome effect on the preservation and sharing of phylogenetic data, there nevertheless remains considerable scope for improvement. The NSF data-management policy, for example, has increased the preservation of alignments but not phylogenetic trees. This is unfortunate, both because phylogenies are more computationally expensive than alignments, and also because most of the reuse of phylogenetic data entails trees rather than sequence alignments [7], [8]. Moreover, although relative archival rates have increased dramatically, the absolute rate remains low: despite recent policy initiatives, a large proportion of datasets are not being captured in online archives. Sustaining the momentum of recent initiatives could be achieved via small measures that increase the benefits and decrease the costs of data sharing to data generators. Although authors who archive data are rewarded with increased citation rates [41], [51], this incentive could be enhanced by rewarding the collection of data as an achievement in its own right. Journal policies can encourage the direct citation of archived datasets in addition to the studies in which the data were generated, and funding agencies and academic institutions can recognize alternative metrics that acknowledge the scientific value of data [52]. Concordantly, the perceived costs of data sharing could be reduced by implementing more flexible embargo policies that protect the priority access of data generators [1], [53].

Clearly, we have a long way to go in order to adequately preserve and freely share phylogenetic data, and the road ahead will not be easy. Nevertheless, our findings suggest that we are moving in the right direction; we are beginning to glimpse the dawn of open access to phylogenetic data.

## Supporting Information

File S1 **Supporting information file describing details of the data collection, data analyses, and results.**
(PDF)

File S2 **Supporting Information file (formatted as a csv table) summarizing the bibliographic data gathered for the 217 studies.** Following [30], this data table has been anonymized to protect the identity of corresponding authors. A key is available upon request from the corresponding author (BRM) to allow details of our analyses to be independently verified.
(CSV)
