# Supplementary material for: The Dawn of Open Access to Phylogenetic Data
Source: PLoS One. 2014 Oct 24;9(10):e110268. doi: 10.1371/journal.pone.0110268 (PMC4208793; doi:10.1371/journal.pone.0110268)
Supplement: File S1 — Supporting information file describing details of the data collection, data analyses, and results. (PDF) [file pone.0110268.s001.pdf]

Supporting Information

Example template messages used for direct solicitation of phylogenetic datasets

Undergraduate First Request

Dear Dr. <corresponding author>,

My name is <solicitor>, and I'm an undergraduate researcher working in the Moore lab at UC Davis. We are doing a meta-analysis of density-dependent rates of lineage diversification. We would like to include your <publication year> study <study title> in our meta-analysis, and were hoping that you could send us any tree files that you used to perform the diversification-rate analysis in your study, as well as the alignment used to generate the study tree.
Thank you very much for your time and consideration.

Sincerely, <solicitor>

Undergraduate Second Request

Dear Dr. <corresponding author>,

I emailed you a few days ago asking for your tree files and alignments from your <publication year> study, <study title>, for inclusion in a meta-analysis of density-dependent lineage diversification. Since I have not yet heard back from you, I would like to reiterate my request for the data, which we would very much like to include.

Sincerely, <solicitor>

Undergraduate Third Request

Dear Dr. <corresponding author>,

I emailed you some time ago asking for your tree file and alignment from your <publication year> study, <study title>, for inclusion in a meta-analysis of density-dependent lineage diversification. I would still appreciate it if you could send me the relevant data.

Sincerely, <solicitor>

Example R script used to generate messages for direct-solicitation campaign

```
table = read.csv("~tree_hunting.csv",sep="\t")

us = c("MRM","AFM","BRM")
invisible(lapply(us,function(PERSON){
    write.table(x=NULL,file=paste("/Tree_hunting/Solicitation
letters/",PERSON,"/",PERSON,".first.letters.txt",sep=""),row.names=FALSE,col.names=FALSE)
}))

apply(table,1,function(study){

    if(study[11] == "Yes"){

        email = as.vector(study[12])
        sender = study[10]

        if(sender == "AFM"){
            file = "/AFM.first.letters.txt"
            text = paste("\nDear Dr. ",study[13],",\n\nMy name is Andy Magee, and I'm an
undergraduate researcher working in the Moore lab at UC Davis. We are doing a meta-analysis of
density-dependent rates of lineage diversification. We would like to include your ",study[2]," study
\"",study[14],"\" in our meta-analysis, and were hoping that you could send us any tree files that
you used to perform the diversification-rate ",ifelse(grepl(".01",study[1]),"analyses","analysis"),"
in your study, as well as the ",ifelse(grepl(".01",study[1]),"alignments","alignment")," used to
generate the study ",ifelse(grepl(".01",study[1]),"trees","tree"),".\n\nThank you very much for your
time and consideration.\n\nSincerely,\nAndy Magee\n",sep="")
        }

        if(sender == "MRM"){
            file = "/MRM.first.letters.txt"
            text = paste("\nDear Dr. ",study[13],",\n\nMy name is Mike May, and I'm a PhD student in
the Moore lab at UC Davis. We are doing a meta-analysis of density-dependent rates of lineage
diversification. We would like to include your ",study[2]," study \"",study[14],"\" in our meta-
analysis, and were hoping that you could send us any tree files that you used to perform the
diversification-rate ",ifelse(grepl(".01",study[1]),"analyses","analysis")," in your study, as well
as the ",ifelse(grepl(".01",study[1]),"alignments","alignment")," used to generate the study
",ifelse(grepl(".01",study[1]),"trees","tree"),".\n\nThank you very much for your time and
consideration.\n\nSincerely,\nMike May\n",sep="")
        }

        if(sender == "BRM"){
            file = "/BRM.first.letters.txt"
            text = paste("\nDear Dr. ",study[13],",\n\nMy name is Brian Moore, and I'm an assistant
professor at UC Davis. I am conducting a meta-analysis of density-dependent rates of lineage
diversification. I would like to include your ",study[2]," study \"",study[14],"\" in my meta-
analysis, and was hoping that you could send me any tree files that you used to perform the
diversification-rate ",ifelse(grepl(".01",study[1]),"analyses","analysis")," in your study, as well
as the ",ifelse(grepl(".01",study[1]),"alignments","alignment")," used to generate the study
",ifelse(grepl(".01",study[1]),"trees","tree"),".\n\nThank you very much for your time and
consideration.\n\nSincerely,\nBrian Moore\n",sep="")
        }

        write.table(x=">>>>>>>>>>>>>>>
\n",file=file,quote=FALSE,sep="",col.names=FALSE,row.names=FALSE,append=TRUE)
        write.table(x=email,file=file,quote=FALSE,sep="",col.names=FALSE,row.names=FALSE,append=TRUE)
        write.table(x=text,file=file,quote=FALSE,sep="",col.names=FALSE,row.names=FALSE,append=TRUE)

    }
})
```

Table S1. Summary of datasets and models.

Name	Source	Data	Predictor variables
$x_{\mathrm{a,a}}$	archived	alignments only	*intercept, age, impact factor, no policy, strong policy, JDAP membership, NSF funding*
$x_{\mathrm{a,t}}$	archived	trees only	*intercept, age, impact factor, no policy, strong policy, JDAP membership, NSF funding*
$x_{\mathrm{a,e}}$	archived	alignments or trees	*intercept, age, impact factor, no policy, strong policy, JDAP membership, NSF funding*
$x_{\mathrm{a,b}}$	archived	alignments and trees	*intercept, age, impact factor, no policy, strong policy, JDAP membership, NSF funding*
$x_{\mathrm{s,a}}$	solicited	alignments only	*intercept, age, impact factor, no policy, strong policy, JDAP membership*, NSF funding*, undergraduate student, professor*
$x_{\mathrm{s,t}}$	solicited	trees only	*intercept, age, impact factor, no policy, strong policy, JDAP membership, NSF funding, undergraduate student, professor*
$x_{\mathrm{s,e}}$	solicited	alignments or trees	*intercept, age, impact factor, no policy, strong policy, JDAP membership, NSF funding, undergraduate student, professor*
$x_{\mathrm{s,b}}$	solicited	alignments and trees	*intercept, age, impact factor, no policy, strong policy, JDAP membership, NSF funding, undergraduate student, professor*
$x_{\mathrm{c,a}}$	combined	alignments only	*intercept, age, impact factor, no policy, strong policy, JDAP membership, NSF funding, solicited*
$x_{\mathrm{c,t}}$	combined	trees only	*intercept, age, impact factor, no policy, strong policy, JDAP membership, NSF funding, solicited*
$x_{\mathrm{c,e}}$	combined	alignments or trees	*intercept, age, impact factor, no policy, strong policy, JDAP membership, NSF funding, solicited*
$x_{\mathrm{c,b}}$	combined	alignments and trees	*intercept, age, impact factor, no policy, strong policy, JDAP membership, NSF funding, solicited*

* – This parameter was initially included in the model, but was later removed because it could not be reliably estimated.

Multicollinearity Analysis

Multicollinearity—complex correlations among predictor variables—can result in pathological parameter estimates in a regression analysis. We used a standard measure of multicollinearity called the *variance inflation factor* (hereafter VIF) to assess the influence of multicollinearity on the results of our logistic regression analyses. We compute the VIF for predictor variable i by performing a linear regression where the value of variable i is treated as the response variable and the values of the remaining variables are treated as the predictor variables. Next, we compute the R^2 value for this linear regression, and then apply the following formula:

$$\mathrm{VIF}_i = \frac{1}{1 - R_i^2}.$$

VIFs represent the relative increase in the variance of the associated parameter estimates caused by unmodeled correlations among predictor variables. A common rule-of-thumb is that VIFs of greater than 4 or 10 are problematic and require correction [53].

We calculated VIFs for each of our predictor variables. For predictor variables that we broke down into sets of dummy variables—namely the *policy* and *solicitor* variables—we used the default dummy variable—*weak policy* and *graduate student*, respectively—for VIF analysis. All of the VIFs were quite small (< 1.5), indicating that multicollinearity is not an issue for our logistic regression analyses (Table S2).

Table S2. Summary of variance inflation factors for each predictor variable.

Predictor variable	VIF
age	1.19
weak policy	1.04
impact factor	1.09
NSF funding	1.25
graduate student	1.01

We also examined pairwise correlations between each predictor variable (summarized in Table S1). Most predictor variables were uncorrelated; however, *NSF funding* and *age* seemed to be correlated, as did *NSF funding* and *impact factor*. The correlation between *NSF funding* and *age* was likely the result of having scored studies for *NSF funding* only if they were published in or after 2011, when NSF instituted its data management policies. The correlation between *NSF funding* and *impact factor* was probably driven by a correlation between quality of research and access to funding. In both cases, the actual correlation coefficients were very small ($\rho = 0.11$ and $\rho = 0.05$, respectively), and so likely had a negligible influence on the results of our logistic regression analysis.

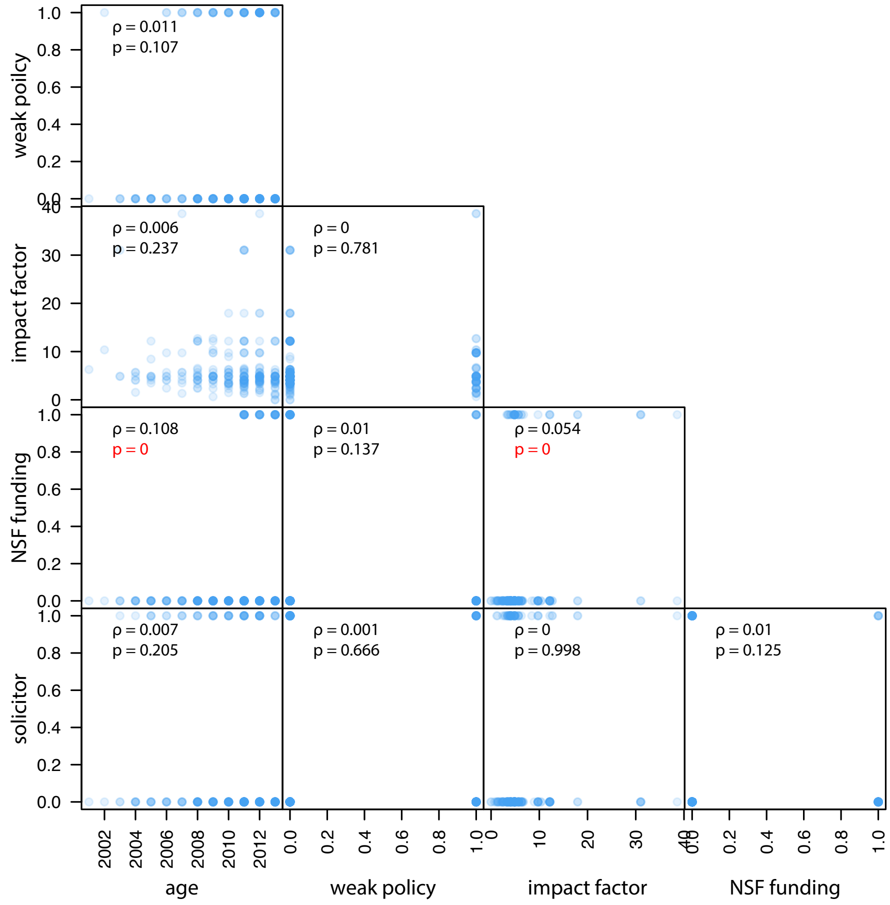

Figure S1. Pairwise correlations between predictor variables. Each panel shows the values of the predictor variable on the x-axis against the values of predictor variable on the y-axis. Correlation coefficients (ρ) and p-values in the top left corner of each panel are from Pearson's product-moment correlations between the predictor variables.

Prior Sensitivity Analysis

To assess the sensitivity of our estimates to the specified priors, we compared estimates of the marginal *posterior* probability density for each parameter to the corresponding marginal *prior* probability density (specified using a standard normal distribution). For each parameter, we computed the Kullback-Leibler (KL) divergence between the marginal prior and posterior densities. The KL divergence measures the distance between two probability distributions, where values close to zero indicate that the distributions are similar [38]. Most of our marginal posterior densities depart strongly from their corresponding prior densities; however, for solicited data, the *JDAP* marginal posterior density is very similar to the prior density (KL divergence ≤ 0.10; see Figures S2, S3).

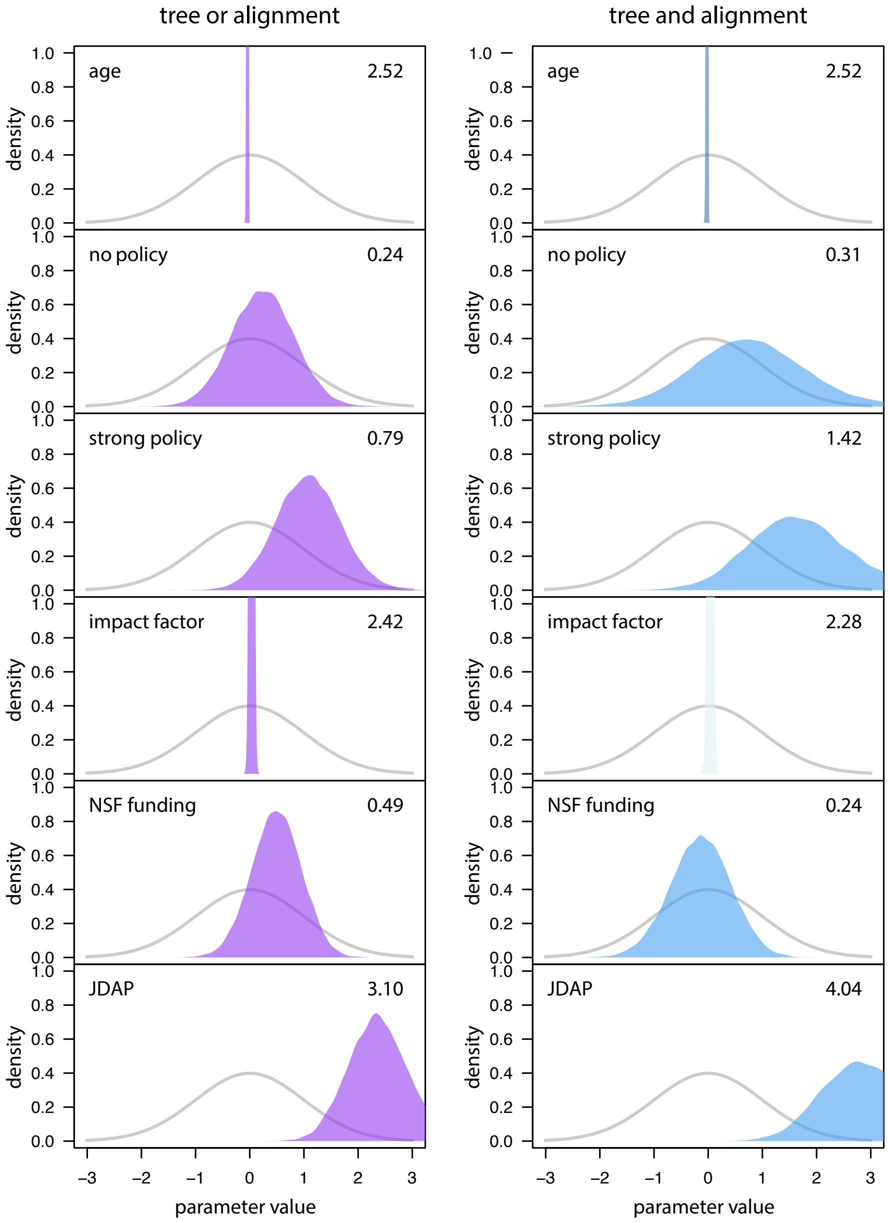

Figure S2. Comparison of marginal posterior and prior densities for archived data. Each panel is a kernel density estimate for a marginal posterior probability density (colored) compared to its prior probability density (grey). The KL divergence for each posterior/prior comparison is indicated in the upper right of each panel; smaller numbers indicate greater similarity. The color scheme of marginal posterior probability densities corresponds to used in Figure 3.

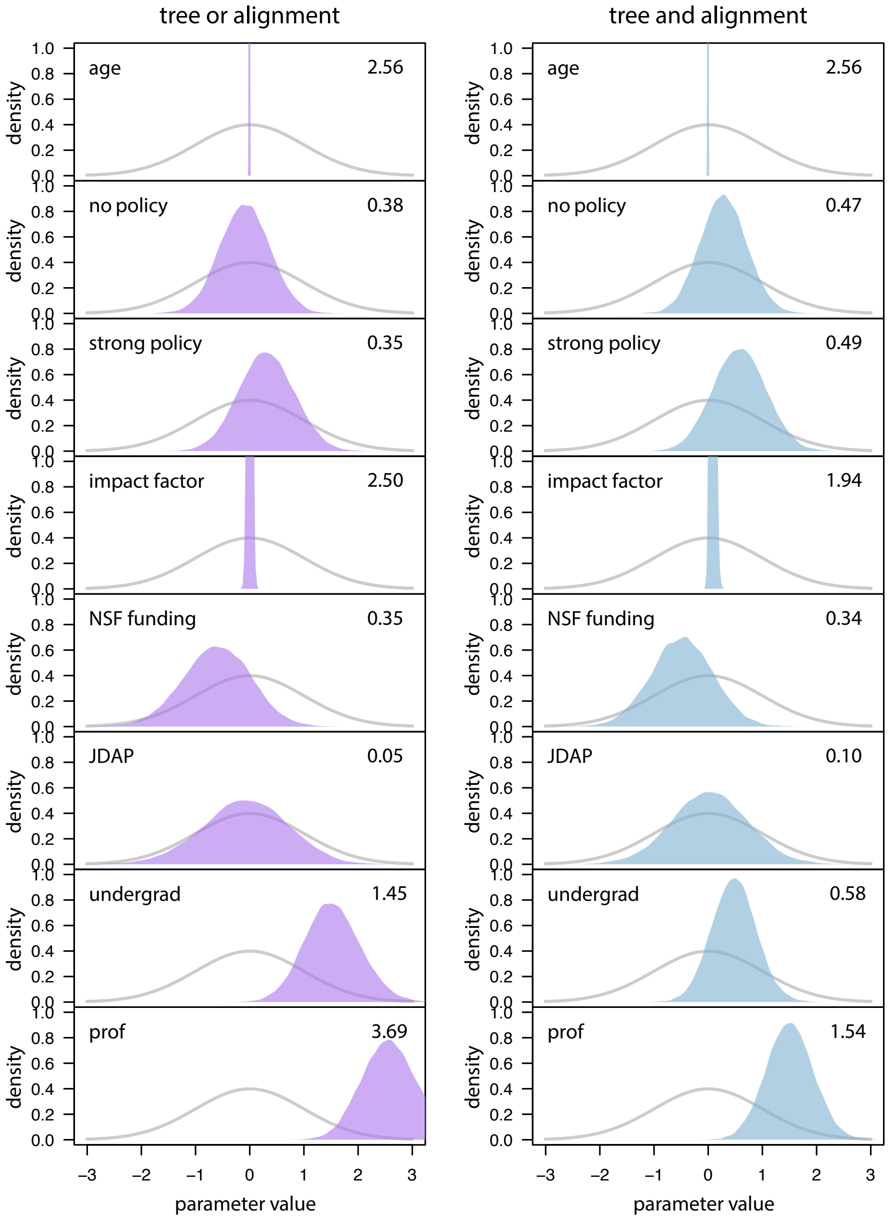

Figure S3. Comparison of marginal posterior and prior densities for solicited data. Each panel is a kernel density estimate for a marginal posterior probability density (colored) compared to its prior probability density (grey). The KL divergence for each posterior/prior comparison is indicated in the upper right of each panel; smaller numbers indicate greater similarity. The color scheme of marginal posterior probability densities corresponds to used in Figure 3.

MCMC diagnosis

Table S3. MCMC performance for analyses of archived alignments or trees.

	Effective Sample Size					Geweke's Diagnostic (p-value)				
Parameter	run 1	run 2	run 3	run 4	combined	run 1	run 2	run 3	run 4	PSRF
β_{I}	7501.00	7501.00	7501.00	7428.18	29931.18	0.36	0.07	0.72	0.64	1.00
β_{age}	7770.81	7501.00	7770.65	6917.10	29959.56	0.74	0.37	0.98	0.64	1.00
β_{IF}	7236.37	7501.00	7501.00	7501.00	29739.37	0.59	0.47	0.11	0.06	1.00
β_{none}	7501.00	7501.00	8372.49	7489.66	30864.15	0.47	0.01	0.68	0.32	1.00
β_{strong}	7501.00	7501.00	7220.13	7501.00	29723.13	0.75	0.55	0.57	0.54	1.00
β_{JDAP}	7501.00	7501.00	7501.00	6773.91	29276.91	0.16	0.18	0.83	0.84	1.00
β_{NSF}	7501.00	7501.00	7964.70	7165.25	30131.94	0.71	0.61	0.55	0.08	1.00

N.B. This corresponds to analysis $x_{a,e}$ in Tables S1 and 1.

Table S4. MCMC performance for analyses of archived alignments and trees.

	Effective Sample Size					Geweke's Diagnostic (p-value)				
Parameter	run 1	run 2	run 3	run 4	combined	run 1	run 2	run 3	run 4	PSRF
β_{I}	7501.00	7501.00	7252.12	7501.00	29755.12	0.29	0.08	0.77	0.21	1.00
β_{age}	7501.00	7501.00	7501.00	7821.37	30324.37	0.26	0.11	0.14	0.86	1.00
β_{IF}	7501.00	6147.95	7501.00	7501.00	28650.95	0.23	0.73	0.03	0.50	1.00
β_{none}	7501.00	7501.00	7501.00	7501.00	30004.00	0.15	0.04	0.29	0.95	1.00
β_{strong}	7616.62	7501.00	7501.00	7501.00	30119.62	0.76	0.03	0.25	0.07	1.00
β_{JDAP}	7501.00	7501.00	7501.00	6887.44	29390.44	0.45	0.08	0.16	0.20	1.00
β_{NSF}	7395.85	7501.00	7501.00	7501.00	29898.85	0.05	0.66	0.31	0.54	1.00

N.B. This corresponds to analysis $x_{a,b}$ in Tables S1 and 1.

Table S5. MCMC performance for analyses of solicited alignments or trees.

	Effective Sample Size					Geweke's Diagnostic (p-value)				
Parameter	run 1	run 2	run 3	run 4	combined	run 1	run 2	run 3	run 4	PSRF
β_{I}	7501.00	7501.00	7501.00	7501.00	30004.00	0.38	0.40	0.58	0.20	1.00
β_{age}	7501.00	7501.00	7501.00	7501.00	30004.00	0.48	0.57	0.68	0.51	1.00
β_{IF}	7501.00	7501.00	7501.00	7501.00	30004.00	0.04	0.58	0.32	0.76	1.00
β_{none}	7501.00	7501.00	7501.00	7501.00	30004.00	0.24	0.28	0.43	0.77	1.00
β_{strong}	7501.00	7501.00	7501.00	7501.00	30004.00	0.63	0.84	0.82	0.73	1.00
β_{JDAP}	7501.00	8065.92	7501.00	7501.00	30568.92	0.87	0.16	0.64	0.39	1.00
β_{NSF}	7501.00	7501.00	7501.00	7383.98	29886.98	0.56	0.87	0.97	0.96	1.00
$\beta_{\mathrm{undergrad}}$	7501.00	7009.73	7501.00	7501.00	29512.73	0.03	0.02	0.58	0.32	1.00
β_{prof}	7501.00	7235.82	7501.00	8881.95	31119.77	0.74	0.68	0.51	0.04	1.00

N.B. This corresponds to analysis $x_{s,e}$ in Tables S1 and 1.

Table S6. MCMC performance for analyses of solicited alignments and trees.

	Effective Sample Size					Geweke's Diagnostic (p-value)				
Parameter	run 1	run 2	run 3	run 4	combined	run 1	run 2	run 3	run 4	PSRF
β_{I}	7501.00	7221.13	8541.62	7501.00	30764.75	0.84	0.33	0.29	0.90	1.00
β_{age}	7501.00	7223.68	7501.00	7501.00	29726.68	0.20	0.98	0.82	0.41	1.00
β_{IF}	7501.00	7501.00	8580.70	7562.06	31144.76	0.90	0.92	0.37	0.31	1.00
β_{none}	7501.00	7501.00	7501.00	7501.00	30004.00	0.09	0.23	0.56	0.17	1.00
β_{strong}	7501.00	7501.00	7501.00	7501.00	30004.00	1.00	0.77	0.62	0.58	1.00
β_{JDAP}	7501.00	7501.00	7501.00	7501.00	30004.00	0.18	0.20	0.69	0.14	1.00
β_{NSF}	7501.00	7501.00	7501.00	7501.00	30004.00	0.97	0.23	0.74	0.10	1.00
$\beta_{\mathrm{undergrad}}$	7185.12	7501.00	7501.00	7501.00	29688.12	0.17	0.93	0.40	0.86	1.00
β_{prof}	7256.96	7501.00	7501.00	7501.00	29759.96	0.28	0.64	0.02	0.04	1.00

N.B. This corresponds to analysis $x_{s,b}$ in Tables S1 and 1.

Table S7. MCMC performance for analyses of archived alignments only.

	Effective Sample Size					Geweke's Diagnostic (p-value)				
Parameter	run 1	run 2	run 3	run 4	combined	run 1	run 2	run 3	run 4	PSRF
β_{I}	7501.00	7501.00	7337.49	7830.03	30169.52	0.10	0.14	0.85	0.21	1.00
β_{age}	6993.23	7501.00	7501.00	7501.00	29496.23	0.24	0.85	0.92	0.62	1.00
β_{IF}	8214.77	7501.00	7501.00	7501.00	30717.77	0.76	0.79	0.43	0.08	1.00
β_{none}	7501.00	7501.00	7501.00	7501.00	30004.00	0.56	0.18	0.74	0.73	1.00
β_{strong}	7501.00	7501.00	7501.00	7501.00	30004.00	0.94	0.24	0.77	0.29	1.00
β_{JDAP}	7501.00	8255.15	7855.49	7501.00	31112.64	0.20	0.03	0.97	0.39	1.00
β_{NSF}	7501.00	7501.00	7501.00	7501.00	30004.00	0.65	0.39	0.49	0.03	1.00

N.B. This corresponds to analysis $x_{a,a}$ in Tables S1 and 1.

Table S8. MCMC performance for analyses of archived trees only.

	Effective Sample Size					Geweke's Diagnostic (p-value)				
Parameter	run 1	run 2	run 3	run 4	combined	run 1	run 2	run 3	run 4	PSRF
β_{I}	7501.00	7501.00	7501.00	7669.34	30172.34	0.66	0.46	0.67	0.44	1.00
β_{age}	7501.00	7092.89	7501.00	7501.00	29595.89	0.74	0.43	0.93	0.02	1.00
β_{IF}	7501.00	7501.00	6660.60	7501.00	29163.60	0.83	0.47	0.85	0.68	1.00
β_{none}	7501.00	7501.00	7501.00	7501.00	30004.00	0.37	0.34	0.17	0.43	1.00
β_{strong}	7501.00	7501.00	7501.00	7501.00	30004.00	0.42	0.73	0.68	0.34	1.00
β_{JDAP}	7501.00	7501.00	7501.00	7068.63	29571.63	0.96	0.63	0.33	0.66	1.00
β_{NSF}	7501.00	7501.00	7501.00	7501.00	30004.00	0.61	0.82	0.63	0.26	1.00

N.B. This corresponds to analysis $x_{a,t}$ in Tables S1 and 1.

Table S9. MCMC performance for analyses of solicited alignments only.

	Effective Sample Size					Geweke's Diagnostic (p-value)				
Parameter	run 1	run 2	run 3	run 4	combined	run 1	run 2	run 3	run 4	PSRF
β_{I}	7501.00	7501.00	7501.00	7501.00	30004.00	0.95	0.31	0.85	0.98	1.00
β_{age}	7501.00	7501.00	7501.00	7501.00	30004.00	0.78	0.38	0.73	0.28	1.00
β_{IF}	7752.87	7788.54	7501.00	7501.00	30543.41	0.81	0.65	0.98	0.81	1.00
β_{none}	7884.20	7501.00	7501.00	7501.00	30387.20	0.38	0.47	0.97	0.78	1.00
β_{strong}	7501.00	7757.03	7501.00	8110.93	30869.96	0.55	0.35	0.62	0.66	1.00
$\beta_{\mathrm{undergrad}}$	7501.00	7501.00	7501.00	8190.22	30693.22	0.77	0.27	0.67	0.73	1.00
β_{prof}	7501.00	7501.00	7501.00	7857.31	30360.31	0.98	0.96	0.96	0.60	1.00

N.B. This corresponds to analysis $x_{s,a}$ in Tables S1 and 1.

Table S10. MCMC performance for analyses of solicited trees only.

	Effective Sample Size					Geweke's Diagnostic (p-value)				
Parameter	run 1	run 2	run 3	run 4	combined	run 1	run 2	run 3	run 4	PSRF
β_{I}	7501.00	7501.00	7501.00	7501.00	30004.00	0.68	0.76	1.00	0.33	1.00
β_{age}	7501.00	7501.00	7501.00	7501.00	30004.00	0.34	0.47	0.92	0.83	1.00
β_{IF}	7501.00	7501.00	7501.00	7501.00	30004.00	0.71	0.24	0.58	0.64	1.00
β_{none}	7229.02	7501.00	7501.00	7806.79	30037.81	0.46	0.34	0.66	0.29	1.00
β_{strong}	7501.00	7501.00	7501.00	7501.00	30004.00	0.92	0.52	0.41	0.13	1.00
β_{JDAP}	7501.00	7501.00	7203.03	7501.00	29706.03	0.34	0.31	0.27	0.10	1.00
β_{NSF}	7501.00	7501.00	7231.46	7501.00	29734.46	0.20	0.71	0.69	0.69	1.00
$\beta_{\mathrm{undergrad}}$	7501.00	7501.00	7501.00	7501.00	30004.00	0.60	0.98	0.58	0.84	1.00
β_{prof}	7429.18	7501.00	7501.00	7501.00	29932.18	0.52	0.97	0.72	0.38	1.00

N.B. This corresponds to analysis $x_{s,t}$ in Tables S1 and 1.

Table S11. MCMC performance for analyses of combined alignments only.

	Effective Sample Size					Geweke's Diagnostic (p-value)				
Parameter	run 1	run 2	run 3	run 4	combined	run 1	run 2	run 3	run 4	PSRF
β_{I}	8932.81	7501.00	7501.00	7501.00	31435.81	0.53	0.38	0.36	0.85	1.00
β_{age}	7501.00	7192.10	7501.00	7203.17	29397.27	0.39	0.61	0.07	0.36	1.00
β_{IF}	7344.84	7501.00	7501.00	7501.00	29847.84	0.61	0.31	0.91	0.38	1.00
β_{none}	7501.00	7904.21	7501.00	7501.00	30407.21	0.47	0.30	0.24	0.11	1.00
β_{strong}	8118.81	7122.63	7501.00	7795.53	30537.97	0.61	0.99	0.07	0.75	1.00
β_{JDAP}	7690.78	7501.00	7501.00	7501.00	30193.78	0.69	0.79	0.12	0.34	1.00
β_{NSF}	7501.00	7501.00	7501.00	7501.00	30004.00	0.53	0.66	0.00	0.76	1.00
$\beta_{\mathrm{solicited}}$	7501.00	7903.44	7501.00	7894.54	30799.98	0.72	0.10	0.05	0.05	1.00

N.B. This corresponds to analysis $x_{c,a}$ in Tables S1 and 1.

Table S12. MCMC performance for analyses of combined trees only.

	Effective Sample Size					Geweke's Diagnostic (p-value)				
Parameter	run 1	run 2	run 3	run 4	combined	run 1	run 2	run 3	run 4	PSRF
β_{I}	7284.35	7501.00	7501.00	7012.23	29298.58	1.00	0.96	0.14	0.38	1.00
β_{age}	6638.97	7501.00	7501.00	7501.00	29141.97	0.97	0.26	0.02	0.69	1.00
β_{IF}	7501.00	7501.00	7716.11	7401.50	30119.61	0.71	0.66	0.96	0.46	1.00
β_{none}	7501.00	7894.56	7501.00	7501.00	30397.56	0.74	0.36	0.98	0.81	1.00
β_{strong}	7501.00	7501.00	6726.40	7501.00	29229.40	0.91	0.89	0.15	0.15	1.00
β_{JDAP}	7501.00	7501.00	8298.20	7233.11	30533.31	0.75	0.22	0.63	0.78	1.00
β_{NSF}	7501.00	7501.00	7501.00	7501.00	30004.00	0.63	0.37	0.39	0.12	1.00
$\beta_{\mathrm{requested}}$	7501.00	7501.00	7501.00	7501.00	30004.00	0.27	0.66	0.23	0.14	1.00

N.B. This corresponds to analysis $x_{c,t}$ in Tables S1 and 1.

Table S13. MCMC performance for analyses of combined alignments or trees.

	Effective Sample Size					Geweke's Diagnostic (p-value)				
Parameter	run 1	run 2	run 3	run 4	combined	run 1	run 2	run 3	run 4	PSRF
β_{I}	6261.86	7501.00	7501.00	6837.35	28101.21	0.25	0.42	0.85	0.45	1.00
β_{age}	7501.00	7501.00	7501.00	7501.00	30004.00	0.10	0.79	0.26	0.29	1.00
β_{IF}	7501.00	7501.00	7501.00	7501.00	30004.00	0.90	0.43	0.27	0.76	1.00
β_{none}	7501.00	7501.00	7501.00	7501.00	30004.00	0.09	0.39	0.71	0.36	1.00
β_{strong}	7501.00	7501.00	7501.00	7501.00	30004.00	0.00	0.18	0.67	0.78	1.00
β_{JDAP}	7521.48	7501.00	7501.00	7240.79	29764.27	0.08	0.57	0.13	0.40	1.00
β_{NSF}	7730.56	7501.00	7501.00	7271.97	30004.53	0.34	0.85	0.40	0.76	1.00
$\beta_{\mathrm{requested}}$	7501.00	7501.00	7501.00	7501.00	30004.00	0.11	0.74	0.35	0.85	1.00

N.B. This corresponds to analysis $x_{c,e}$ in Tables S1 and 1.

Table S14. MCMC performance for analyses of combined alignments and trees.

Parameter	Effective Sample Size					Geweke's Diagnostic (p-value)				
	run 1	run 2	run 3	run 4	combined	run 1	run 2	run 3	run 4	PSRF
β_{I}	7501.00	7501.00	7501.00	7501.00	30004.00	0.44	0.38	0.62	0.75	1.00
β_{age}	7501.00	7501.00	7501.00	7501.00	30004.00	0.51	0.09	0.46	0.22	1.00
β_{IF}	7501.00	7501.00	7501.00	7501.00	30004.00	0.59	0.56	0.14	0.79	1.00
β_{none}	8623.45	7501.00	7501.00	7841.88	31467.33	0.77	0.72	0.44	0.91	1.00
β_{strong}	7501.00	7501.00	7501.00	7501.00	30004.00	0.18	0.47	0.34	0.27	1.00
β_{JDAP}	7918.18	7501.00	7501.00	7281.23	30201.41	0.16	0.35	0.78	0.65	1.00
β_{NSF}	7501.00	7501.00	7772.42	8128.34	30902.75	0.71	0.29	0.28	0.23	1.00
$\beta_{\mathrm{requested}}$	7758.05	7501.00	7240.75	7501.00	30000.80	0.45	0.89	0.72	0.75	1.00

N.B. This corresponds to analysis $x_{c,a}$ in Tables S1 and 1.

Logistic model parameter estimates

Table S15. Parameter estimates based on archived data.

	alignments only			trees only			alignments or trees			alignments and trees		
		95% HPD			95% HPD			95% HPD			95% HPD	
	mean	lower	upper	mean	lower	upper	mean	lower	upper	mean	lower	upper
β_{I}	-3.114	-5.028	-1.266	-0.864	-2.623	0.627	-0.968	-1.958	0.067	-3.172	-5.009	-1.459
β_{age}	-0.033	-0.069	0.002	-0.080	-0.139	-0.026	-0.038	-0.060	-0.016	-0.019	-0.043	0.003
β_{IF}	-0.018	-0.127	0.084	0.035	-0.068	0.141	0.036	-0.028	0.097	0.045	-0.028	0.118
β_{none}	0.650	-1.357	2.761	-0.627	-2.615	1.310	0.234	-0.886	1.386	0.783	-1.274	2.883
β_{strong}	1.001	-1.023	3.293	-0.082	-2.323	1.903	1.075	-0.044	2.284	1.722	-0.087	3.712
β_{JDAP}	1.685	-0.114	3.594	0.066	-1.529	1.791	2.370	1.382	3.528	3.048	1.289	4.854
β_{NSF}	1.183	-0.045	2.446	-0.259	-1.769	1.290	0.498	-0.355	1.393	-0.143	-1.234	0.928

Table S16. Parameter estimates based on solicited data.

	alignments only			trees only			alignments or trees			alignments and trees		
		95% HPD			95% HPD			95% HPD			95% HPD	
	mean	lower	upper	mean	lower	upper	mean	lower	upper	mean	lower	upper
β_{I}	-6.248	-9.587	-3.130	-1.577	-3.096	-0.248	-0.328	-1.225	0.548	-1.708	-2.780	-0.619
β_{age}	0.004	-0.018	0.027	0.001	-0.018	0.022	-0.004	-0.016	0.007	-0.006	-0.019	0.005
β_{IF}	-0.338	-0.733	0.024	0.130	0.034	0.222	0.081	-0.003	0.171	0.002	-0.076	0.075
β_{none}	0.653	-1.530	2.787	0.578	-0.953	2.062	0.281	-0.599	1.127	-0.089	-1.013	0.805
β_{strong}	1.555	-0.622	3.810	0.209	-1.466	1.876	0.573	-0.425	1.499	0.311	-0.661	1.303
β_{JDAP}	NA	NA	NA	0.622	-1.617	2.863	0.027	-1.424	1.347	-0.095	-1.609	1.469
β_{NSF}	NA	NA	NA	0.220	-1.538	1.907	-0.482	-1.590	0.663	-0.612	-1.901	0.556
$\beta_{\text{undergrad}}$	2.177	-0.388	4.978	-2.736	-4.622	-0.960	0.478	-0.356	1.231	1.538	0.602	2.598
β_{prof}	1.507	-1.153	4.508	-2.334	-3.991	-0.819	1.501	0.653	2.378	2.598	1.649	3.645

Table S17. Parameter estimates based on archived and solicited data.

	alignments only			trees only			alignments or trees			alignments and trees		
		95% HPD			95% HPD			95% HPD			95% HPD	
	mean	lower	upper	mean	lower	upper	mean	lower	upper	mean	lower	upper
β_{I}	-0.994	-1.887	-0.082	0.479	-1.453	2.380	2.111	1.063	3.180	-0.171	-1.389	0.953
β_{age}	-0.008	-0.019	0.002	-0.038	-0.078	0.002	-0.007	-0.018	0.002	0.006	-0.009	0.021
β_{IF}	-0.005	-0.060	0.049	0.004	-0.102	0.111	0.002	-0.054	0.059	-0.012	-0.083	0.058
β_{none}	-0.114	-0.867	0.679	-0.335	-2.227	1.589	0.101	-0.688	0.834	0.772	-0.445	1.992
β_{strong}	0.289	-0.548	1.084	-0.609	-2.517	1.313	0.338	-0.477	1.171	0.353	-0.814	1.611
β_{JDAP}	-0.033	-1.066	0.916	-1.334	-3.174	0.506	0.431	-0.656	1.513	1.238	0.010	2.467
β_{NSF}	0.232	-0.593	1.074	-0.858	-2.429	0.663	-0.342	-1.235	0.520	0.172	-0.880	1.211
$\beta_{\text{requested}}$	0.690	-0.098	1.438	-3.634	-5.544	-1.835	-2.040	-2.948	-1.132	-3.162	-4.099	-2.239

Journal Policies

The American Naturalist **(strong policy, JDAP member)**

The American Naturalist *requires authors to deposit the data associated with accepted papers in a public archive.* For gene sequence data and phylogenetic trees, deposition in GenBank or TreeBASE, respectively, is required. There are many possible archives that may suit a particular data set, including the Dryad repository for ecological and evolutionary biology data (http://datadryad.org). All accession numbers for GenBank, TreeBASE, and Dryad must be included in accepted manuscripts before they go to Production. If the data are deposited somewhere else, please provide a link. If the data are culled from published literature, please deposit the collated data in Dryad for the convenience of your readers. Any impediments to data sharing should be brought to the attention of the editors at the time of submission so that appropriate arrangements can be worked out. For more, see the editorial on data.

Annals of Botany **(weak policy)**

Before novel sequences for proteins or nucleotides can be published, authors are required to deposit their data with one of the principal databases comprising the International Nucleotide Sequence Database Collaboration: EMBL Nucleotide Sequence Database, GenBank, or the DNA Data Bank of Japan and to include an accession number in the paper.

Sequence matrices should only be included if alignment information is critical to the message of the paper. Such matrices can be in colour but should not occupy more than one printed page. Larger matrices will only be printed by special agreement but may more readily be published electronically as Supplementary Information.

Australian Systematic Botany **(weak policy)**

For all papers, whether presenting morphological, cytological or molecular data, voucher specimens must be cited, along with the herbarium where lodged.

All sequences used as data must be deposited in one of the international nucleotide sequence databases, preferably GenBank, National Center for Biotechnology Information, 8600 Rockville Pike, Bethesda, MD 20894, USA. Email: gb-sub@ncbi.nlm.nih.gov. Request information at gsdb@gsdb.ncgr.org. Post-review final manuscript will not be accepted until sequence database accession numbers are included.

Biological Journal of the Linnean Society **(weak policy†)**

Data that are integral to the paper must be made available in such a way as to enable readers to replicate, verify and build upon the conclusions published in the paper. Any restriction on the availability of these data must be disclosed at the time of submission. Data may be included as part of the main article where practical. We *recommend that data for which public repositories are widely used, and are accessible to all, should be deposited in such a repository prior to publication.* The appropriate linking details and identifier(s) should then be included in the publication and where possible the repository, to facilitate linking between the journal article and the data. If such a repository does not exist, data should be included as supporting information to the published paper or authors should agree to make their data available upon reasonable request.

NB: †Data-sharing policy in place at the time of study publication, which has since become *JDAP membership.*

BMC Biology **(weak policy)**

Submission of a manuscript to a BioMed Central journal implies that *readily reproducible materials described in the manuscript, including all relevant raw data, will be freely available to any scientist wishing to use them* for non-commercial purposes.

Through a special arrangement with LabArchives, LLC, authors submitting manuscripts to BMC Biology can obtain a complimentary subscription to LabArchives with an allotment of 100MB of storage. LabArchives is an Electronic Laboratory Notebook which will enable scientists to share and publish data files in situ; you can then link your paper to these data. Data files linked to published articles are assigned digital object identifiers (DOIs) and will remain available in perpetuity. Use of LabArchives or similar data publishing services does not replace preexisting data deposition requirements, such as for nucleic acid sequences, protein sequences and atomic coordinates.

The Accession Numbers of any nucleic acid sequences, protein sequences or atomic coordinates cited in the manuscript should be provided, in square brackets and include the corresponding database name

BMC Evolutionary Biology **(no policy†)**

BMC Evolutionary Biology *encourages* authors to deposit the data set(s) supporting the results reported in submitted manuscripts in a publicly-accessible data repository, when it is not possible to publish them as additional files. This section should only be included when supporting data are available and must include the name of the repository and the permanent identifier or accession number and persistent

hyperlink(s) for the data set(s).

NB: †Data-sharing policy in place at the time of study publication, which has since become *JDAP membership.*

***BMC Plant Biology* (weak policy)**

The Accession Numbers of any nucleic acid sequences, protein sequences or atomic coordinates cited in the manuscript should be provided, in square brackets and include the corresponding database name· · ·

The databases for which we can provide direct links are: EMBL Nucleotide Sequence Database (EMBL), DNA Data Bank of Japan (DDBJ), GenBank at the NCBI (GenBank), Protein Data Bank (PDB), Protein Information Resource (PIR) and the Swiss-Prot Protein Database (Swiss-Prot).

BMC Plant Biology encourages authors to deposit the data set(s) supporting the results reported in submitted manuscripts *in a publicly-accessible data repository,* when it is not possible to publish them as additional files. This section should only be included when supporting data are available and must include the name of the repository and the permanent identifier or accession number and persistent hyperlink(s) for the data set(s).

Through a special arrangement with LabArchives, LLC, authors submitting manuscripts to BMC Plant Biology can obtain a complimentary subscription to LabArchives with an allotment of 100MB of storage. LabArchives is an Electronic Laboratory Notebook which will enable scientists to share and publish data files in situ; you can then link your paper to these data. Data files linked to published articles are assigned digital object identifiers (DOIs) and will remain available in perpetuity. Use of LabArchives or similar data publishing services does not replace preexisting data deposition requirements, such as for nucleic acid sequences, protein sequences and atomic coordinates.

***Cladistics* (weak policy)**

Cladistics requests the deposition of data matrices and other material electronically *for publication on the Willi Hennig Society Journal web site.* Please submit these data as e-mail attachments to the Associate Editor Mark Siddall (siddall@amnh.org) after receiving the tracking number for your manuscript from the Editor. These data will be made available to the referees but not to the community at large until such time as the paper is accepted. If the paper is not found to be acceptable for publication, the data and associated files will be removed from the directory structure and destroyed.

***Copeia* (weak policy)**

Analyses based on molecular sequence data must cite the relevant GenBank accession numbers in the text.

Ecology **(no policy†)**

The editors and publisher expect authors to make the data underlying published articles available. Although *public data availability is not strictly a requirement for manuscripts published in Ecology*, Ecological Applications and Ecosphere at this time, any information on materials, methods or data necessary to verify the conclusions of the research reported must be made available to the Subject-matter Editor upon request.

NB: †Data-sharing policy in place at the time of study publication, which has since become *JDAP membership.*

Ecology and Evolution **(strong policy)**

The Journal Ecology and Evolution *requires, as a condition for publication, that data supporting the results in the paper should be archived in an appropriate public archive,* such as GenBank, TreeBASE, Dryad, the Knowledge Network for Biocomplexity or other suitable long-term and stable public repositories. Data are important products of the scientific enterprise, and they should be preserved and usable for decades in the future.

Ecology Letters **(no policy)**

It is *recommended that authors deposit the data supporting the results* in the paper in a publicly accessible archive, such as Dryad (DataDryad.Org). Data are important products of scientific enterprise, and they should be preserved and remain usable in future decades. DNA sequences published in Ecology Letters should be deposited in the EMBL/GenBank/DDJB Nucleotide Sequence Databases. An accession number for each sequence must be included in the manuscript.

Evolution **(strong policy, JDAP membership)**

As a condition for publication, Evolution *requires that data used in the paper are archived. DNA sequence data must be submitted to GenBank and phylogenetic data to TreeBASE. Other types of data must be deposited in an appropriate public archive* such as Dryad, the NCEAS Data Repository, or as supplementary online material associated with the paper published in Evolution. *The data should be given with sufficient detail that, together with the contents of the paper, they allow each result in the published paper to be recreated.* Authors may elect to have the data publicly available at time of publication, or,

if the technology of the archive allows, may opt to embargo access to the data for a period up to a year after publication. Exceptions may be granted at the discretion of the Editor-in-Chief, especially for sensitive information such as the location of endangered species. Authors must state their intention to archive their data when they submit their manuscript and must confirm that this has been done before the manuscript is sent to press. If a repository is to be cited, the citation should include the sequence name and accession number, if available. The basic format for citing electronic resources is: Author's Last Name, First initial. Title of data package (e.g., Data from Article name). Data Repository Name, Data identifier (or DOI), address/URL. Please include on your title page the location of your data or where you intend to archive your data.

Evolutionary Bioinformatics Online **(weak policy)**

Authors publishing in Libertas Academica journals must agree to *make freely available to other academic researchers any of the cells, clones of cells, DNA, antibodies, or other material used in the research reported and not available from commercial suppliers.*

Use DNA Databank of Japan, European Molecular Biology Laboratory, or GenBank.

Please *ensure that accession numbers of any nucleaic acid sequences, protein sequences or atomic coordinates cited in the manuscript are provided* in square brackets with the corresponding database name.

Generally it is possible to provide direct links to data hosted on these databases: EMBL Nucleotide Sequence Database, DNA Data Bank of Japan, GenBank at the NCBI, Protein Data Bank, Protein Information Resource and the SwissProt Protein Database.

Evolutionary Biology **(weak policy)**

DNA sequences must be submitted to GenBank (NCBI - National Center for Biotechnology Information, Bethesda, USA) *or to the EMBL Nucleotide Sequence Data Base* (EBI - European Institute of Bioinformatics, Cambridge, UK) and *accession numbers must be provided* when the paper is accepted.

Authors are encouraged to submit other data types to online data sharing resources if such resources are available.

Hydrobiologia **(no policy)**

International Journal of Plant Sciences **(no policy)**

Material that is not integral to the body of the article and that substantially lengthens the print version of the article (e.g., genetic and character matrices, *extended cladograms*, extended tables) *should be desgignated as appendixes* (table A1, etc.) and thus appear in the electronic edition of IJPS only. Exception: *If voucher material is presented in your manuscript, this should be listed in the first appendix* (appendix A1), and this will appear in the print version of IJPS. To prepare your accession data, provide an appendix title and a sentence-style row of headings for the data. For each taxon sampled, include specimen voucher information and/or gene accession numbers, separated by commas. To save space, the taxa should be run together in a paragraph.

***ISME Journal* (strong policy)**

The ISME Journal will *only review and publish manuscripts if the authors agree to make all data that cannot be published in the journal itself* (e.g. novel nucleotide sequences, structural data, or data from large-scale gene expression experiments) *freely available in one of the public databases* (see Submission to public databases below). *Accession codes must be provided* at the time a revised manuscript is returned to the Editorial Office. To avoid delays in publication of the manuscript, *we encourage authors to deposit relevant data in public databases prior to submission.*

***Italian Journal of Zoology* (no policy)**

***Journal of Arid Environments* (no policy)**

Elsevier *accepts electronic supplementary material to support and enhance* your scientific research. Supplementary files offer the author additional possibilities to publish supporting applications, high-resolution images, background datasets, sound clips and more. Supplementary files supplied will be published online alongside the electronic version of your article in Elsevier Web products, including ScienceDirec

Electronic archiving of supplementary data enables readers to replicate, verify and build upon the conclusions published in your paper. We *recommend that data should be deposited* in the data library PANGAEA

***Journal of Biogeography* (weak policy)**

Consistent with widely adopted conventions in the field, it is a condition of publication that *papers using new molecular sequences must place the sequences in an appropriate database* (e.g. GenBank). Relevant accession numbers should be provided in the final manuscript. *Accession numbers are required for all*

sequences used in analyses, including existing sequences in databases. Museum voucher numbers should also be provided where doing so constitutes the appropriate best practice and/or where this information could be of real value to future researchers. More generally, the journal recognizes that what is considered appropriate best practice regarding data publication/deposition may vary depending on factors such as the nature of the data, the funding sources involved, complexities of prior intellectual ownership issues, etc. We therefore *strongly encourage (where appropriate) but do not require (where it may not be) authors to publish/deposit data sets* in conjunction with papers being published in this journal.

Authors who wish to provide a consolidated statement of how other readers can access the data used in their paper *may wish to refer to outside data repositories* where they have deposited their data, e.g. Dryad, Pangaea, or others. If so, this statement should be included after the Supporting Information section and before the Biosketch entry.

Additional materials and results (including supporting tables and figures) that are necessary but do not need to be included in the main paper must be compiled into Appendices, which will be provided to readers as online Supporting Information. No more than three supplementary appendices are permitted (labelled Appendix S1 to Appendix S3).

Journal of Evolutionary Biology **(strong policy, JDAP membership)**

Submission of a manuscript to a BioMed Central journal implies that readily reproducible materials described in the manuscript, including *all relevant raw data, will be freely available to any scientist wishing to use them for non-commercial purposes.*

The Journal of Evolutionary Biology requires, as a condition for publication, that *data supporting the results in the paper should be archived in an appropriate public archive,* such as GenBank, TreeBASE, Dryad, the Knowledge Network for Biocomplexity or other suitable long-term and stable public repositories. Data are important products of the scientific enterprise, and they should be preserved and usable for decades in the future.

All accepted papers should *provide accession numbers or DOI* for data underlying the work that have been deposited, so that these can appear in the final accepted article.

Journal of Heredity **(strong policy[†])**

The primary data underlying the conclusions of an article are critical to the verifiability and transparency of the scientific enterprise, and *should be preserved in usable form for decades in the future.* For this reason,

Journal of Heredity has *adopted the Joint Data Archiving Policy* (JDAP)(See Editorial, J Hered (2013) 104 (1):1. doi: 10.1093/jhered/ess137). *The public archiving of all primary data is a requirement* of publication in Journal of Heredity. For data other than nucleotide and protein sequences submitted to GenBank, *suitable archives include Dryad, TreeBASE* or the Knowledge Network for Biocomplexity. *For accepted articles, JHered covers the cost to archive the data in Dryad.*

Authors may elect to have the data publicly available at time of publication, or may opt to embargo access to the data for a period up to a year after publication. Exceptions may be granted at the discretion of the Editor, especially for sensitive information, such as the location of endangered species.
NB: †Data-sharing policy in place at the time of study publication, which has since become *JDAP membership.*

Journal of Mammalian Evolution **(no policy)**

Authors submitting analyses of gene or amino acid sequences should be prepared to supply electronic versions of their sequence alignments *if requested by reviewers.* These are for confidential examination by reviewers only, and reviewers are requested not to use these alignments for any purpose other than the review process.

If the reported research includes examination of *voucher specimens (including tissues), the Museum catalogue number or tissue number if the former is not available must be provided.* If sequences from Genbank are used, the *Museum catalogue number* listed on Genbank must be listed, as well as the Genbank accession number. Genbank accession numbers can be used alone, only in the event that the Museum catalogue number is not available through the Genbank record.

Journal of Mammology **(weak policy)**

All DNA sequences must be submitted to GenBank, and accession numbers provided in the manuscript before publication.

Supplemental les will be posted online-only and provides information that adds depth to a manuscript but is not essential to a readers understanding of the research (e.g., spreadsheets, databases, equations, video or audio files, tables and/or figures).

Journal of Theoretical Biology **(no policy)**

Elsevier *encourages authors to connect articles with external databases*, giving their readers one-click

access to relevant databases that help to build a better understanding of the described research. Please refer to relevant database identifiers using the following format in your article

Elsevier *accepts electronic supplementary material* to support and enhance your scientific research. Supplementary files offer the author additional possibilities to publish supporting applications, high-resolution images, background datasets, sound clips and more. Supplementary files supplied will be published online alongside the electronic version of your article in Elsevier Web products, including ScienceDirect

Methods in Ecology and Evolution **(strong policy)**

Data are important products of the scientific enterprise, and they should be preserved and usable for decades in the future. The British Ecological Society thus *expects that data* (or, for theoretical papers, mathematical and computer models) *supporting the results in Methods in Ecology and Evolution papers will be archived in an appropriate public archive, such as Dryad, TreeBASE,* NERC data centre, GenBank, or another archive of the author's choice that provides comparable access and guarantee of preservation.

Sufficient details should be archived so that a third party can reasonably interpret those data correctly, *to allow each result in the published paper to be recreated and the analyses reported in the paper to be replicated* to support the conclusions made. *Authors are welcome to archive more than this, but not less.*

If data have been previously archived then they should not be archived again. *The original archive DOI or reference should be used as the source of the data.*

Molecular Biology and Evolution **(weak policy†)**

Among the *requirements for publication* in MBE is that *authors make publicly available, free of charge, any alignment data*, strains, cell lines, or clones used in reported experiments, computer code essential to the analysis, *and any other material or information necessary for the assessment and verification of findings* or interpretations presented in the publication.

Newly reported sequences must be deposited in the DDBJ/EMBL/GenBank database (see below). *Accession numbers must be included in the final version of the manuscript* and cannot be added at the proof stage.

Newly reported nucleic acid and amino acid sequences, microarray data, structural coordinates, and all other essential information must be submitted to appropriate public databases (e.g., GenBank; the EMBL Nucleotide Sequence Database; DNA Database of Japan; the Protein Data Bank; Swiss-Prot;

GEO; and Array-Express).

When appropriate, material such as sequence alignments and large tables *can be published online as supplementary material* permanently linked to an article in the online journal.

NB: †Data-sharing policy in place at the time of study publication, which has since become *JDAP membership.*

Molecular Ecology **(strong policy, JDAP membership)**

Molecular Ecology expects that *data supporting the results in the paper should be archived in an appropriate public archive,* such as *GenBank*, Gene Expression Omnibus, *TreeBASE, Dryad,* the Knowledge Network for Biocomplexity, your own institutional or funder repository, or as Supporting Information on the Molecular Ecology web site. Data are important products of the scientific enterprise, and they should be preserved and usable for decades in the future.

Authors are expected to archive the data supporting their results and conclusions along with sufficient details so that a third party can interpret them correctly.

To enable readers to locate archived data from Molecular Ecology papers, *we require that authors include a Data Accessibility section* after the references (see below for details). This section must be present at initial submission.

Molecular Phylogenetics and Evolution **(no policy)**

Elsevier *encourages authors to connect articles with external databases,* giving their readers one-click access to relevant databases that help to build a better understanding of the described research.

Elsevier *accepts* electronic supplementary *material to support and enhance* your scientific research. Supplementary files offer the author additional possibilities to publish supporting applications, high-resolution images, background datasets, sound clips and more. Supplementary files supplied will be published online alongside the electronic version of your article in Elsevier Web products, including ScienceDirect: http://www.sciencedirect.com.

You can *enrich* your online articles *by providing phylogenetic tree data files (optional)* in Newick or NeXML format, which will be visualized using the interactive tree viewer embedded within the online article. Using the viewer it will be possible to zoom into certain tree areas, change the tree layout, search within the tree, and collapse/expand tree nodes and branches. Submitted tree files will also be available for downloading from your online article on ScienceDirect. Each tree must be contained in an individual

data file before being uploaded separately to the online submission system, via the 'phylogenetic tree data' submission category. Newick files must have the extension .new or .nwk (note that a semicolon is needed to end the tree). Please do not enclose comments in Newick files and also delete any artificial line breaks within the tree data because these will stop the tree from showing. For NeXML, the file extension should be .xml. Please do not enclose comments in the file. Tree data submitted with other file extensions will not be processed. Please make sure that you validate your Newick/NeXML files prior to submission. For more information please see http://www.elsevier.com/phylogenetictrees.

Nature **(weak policy†)**

Therefore, a condition of publication in a Nature journal is that *authors are required to make materials, data and associated protocols promptly available to readers* without undue qualifications.

The preferred way to share large data sets is via public repositories.

NB: †Data-sharing policy in place at the time of study publication, which has since become *JDAP membership.*

New Phytologist **(weak policy)**

Perspectives in Plant Ecology, Evolution and Systematics **(no policy)**

Elsevier *accepts electronic supplementary material to support and enhance* your scientific research. Supplementary files offer the author additional possibilities to publish supporting applications, high-resolution images, background datasets, sound clips and more.

You can *enrich your online articles* by providing phylogenetic tree data files (optional) in Newick or NeXML format, which will be visualized using the interactive tree viewer embedded within the online article.

Electronic archiving of supplementary data enables readers to replicate, verify and build upon the conclusions published in your paper. We *recommend that data should be deposited* in the data library PANGAEA (http://www.pangaea.de).

Plant Systematics and Evolution **(weak policy)**

Data matrices *including sequence alignments must be made available to the public.* There must be a sentence included in the Materials and methods section that such information is available from the

corresponding author. *DNA or proteine sequences must be deposited in public data bases* (GenBank, EMBL, etc.) before the revised version is sent to the editor.

***PLOS ONE* (weak policy†)**

Manuscripts reporting paleontology and archaeology research must include descriptions of methods and specimens in sufficient detail to allow the work to be reproduced. *Data sets supporting statistical and phylogenetic analyses should be provided, preferably in a format that allows easy re-use.*

Specimen numbers and complete repository information, including museum name and geographic location, *are required for publication.* Locality information should be provided in the manuscript as legally allowable, or a statement should be included giving details of the availability of such information to qualified researchers.

Methods sections of papers with data that should be deposited in a publicly available database *should specify where the data have been deposited* and provide the relevant accession numbers and version numbers, if appropriate. Accession numbers should be provided in parentheses after the entity on first use. If the accession numbers have not yet been obtained at the time of submission, please state that they will be provided during review. They must be provided prior to publication.

NB: †Data-sharing policy in place at the time of study publication, which has since become *JDAP membership.*

***PLOS Biology* (weak policy†)**

The results section should provide details of all of the experiments that are required to support the conclusions of the paper.

Large datasets, including raw data, should be submitted as supplemental files; these are published online alongside the accepted article.

NB: †Data-sharing policy in place at the time of study publication, which has since become *JDAP membership.*

***Proceedings of the National Academy of Sciences* (weak policy)**

To allow others to replicate and build on work published in PNAS, *authors must make materials, data, and associated protocols available to readers.* Authors must disclose upon submission of the manuscript any restrictions on the availability of materials or information. Data not shown and personal communications

cannot be used to support claims in the work. Authors are encouraged to use SI to show all necessary data. Authors are encouraged to deposit as much of their data as possible in publicly accessible databases.

Before publication, *authors must deposit large datasets (including microarray data, protein or nucleic acid sequences, and atomic coordinates for macromolecular structures) in an approved database* and provide an accession number for inclusion in the published paper. *When no public repository exists, authors must provide the data as SI online* or, in special circumstances when this is not possible, on the authors institutional Web site, provided that a copy of the data is provided to PNAS.

Authors must deposit data in a publicly available database such as GenBank, EMBL, DNA Data Bank of Japan, UniProtKB/Swiss-Prot, or PRIDE.

***Proceedings of the Royal Academy B: Biological Sciences* (strong policy)**

To allow others to verify and build on the work published in Royal Society journals *it is a condition of publication that authors make available the data and research materials supporting the results in the article,* as detailed in our Publishing policies; with which our authors are asked to comply.

Datasets should be deposited in an appropriate, recognized repository and the associated accession number, link or DOI to the datasets must be included in the methods section of the article. Reference(s) to datasets should also be included in the reference list of the article with DOIs (where available). Where no discipline-specific data repository exists authors should deposit their datasets in a general repository such as Dryad (http://datadryad.org/)

Where possible any other relevant research materials (such as statistical tools, protocols, software etc) should also be made available and details of how they may be obtained should be included in the data accessibility section of the article.

To ensure archived data from Proceedings B articles are available to readers, *authors should include a data accessibility section* immediately after the acknowledgements. This should list the database and accession number for all data from the article that has been made publicly available.

***Science* (strong policy)**

All data necessary to understand, assess, and extend the conclusions of the manuscript must be available to any reader of Science. All computer codes involved in the creation or analysis of data must also be available to any reader of Science. *After publication, all reasonable requests for data and materials must be fulfilled.*

Science supports the efforts of databases that aggregate published data for the use of the scientific community. Therefore, *appropriate data sets (including microarray data, protein or DNA sequences, atomic coordinates or electron microscopy maps for macromolecular structures, and climate data) must be deposited in an approved database, and an accession number or a specific access address must be included in the published paper.* We encourage compliance with MIBBI guidelines (Minimum Information for Biological and Biomedical Investigations).

Large data sets with no appropriate approved repository must be housed as supplementary materials at Science, or only when this is not possible, on an archived institutional Web site, provided a copy of the data is held in escrow at Science to ensure availability to readers.

Systematic Biology **(strong policy)**

All nucleotide sequence data and alignments must be submitted to GenBank or EMBL before the paper can be published. In addition, *all data matrices and resulting trees must be submitted to TreeBASE.* GenBank and TreeBASE *reference numbers should be provided in the final version of the paper.*

Systematic Entomology **(no policy)**

Paper submissions will be accepted exceptionally, although any relevant data matrices should be electronic.

Trends in Microbiology **(no policy)**

A Trends in Microbiology Reviews *must not include unpublished data, simulations or meta-analyses,* or propose a new formal mathematical model.

A Trends in Microbiology Opinion *must not include unpublished data, simulations or meta-analyses,* or propose a new formal mathematical model.

Science Society articles *should not include unpublished data, simulations or meta-analyses.*

Letters should *not be used as an opportunity* to promote your own work, *to introduce new data,* to provide an 'update' on a recent review, or to highlight perceived omissions in our articles.

Virus Research **(no policy)**

Elsevier *encourages authors to connect articles with external databases,* giving their readers one-click access to relevant databases that help to build a better understanding of the described research.

Elsevier *accepts electronic supplementary material to support and enhance your scientific research.* Supplementary files offer the author additional possibilities to publish supporting applications, high-resolution images, background datasets, sound clips and more. Supplementary files supplied will be published online alongside the electronic version of your article in Elsevier Web products, including ScienceDirec

***Zoological Journal of the Linnean Society* (no policy)**

Avoid elaborate tables of original or derived data, long lists of species, etc.; *if* such data are *absolutely essential,* consider including them as appendices or as online-only supplementary material.

Table S18. Summary of journal policies.

Journal	Policy rating	Number of studies
The American Naturalist	*JDAP membership*	5
Annals of Botany	*weak policy*	1
Australian Systematic Botany	*weak policy*	2
Biological Journal of the Linnean Society†	*weak policy*	4
Biogeography	*weak policy*	15
BMC Biology	*weak policy*	3
BMC Evolutionary Biology†	*no policy*	12
BMC Plant Biology	*weak policy*	1
Cladistics	*weak policy*	1
Copeia	*weak policy*	1
Ecology†	*no policy*	2
Ecology and Evolution	*strong policy*	2
Ecology Letters	*no policy*	4
Evolution	*JDAP membership*	35
Evolutionary Biology	*weak policy*	1
Hydrobiologia	*no policy*	1
International Journal of Plant Sciences	*no policy*	1
International Journal of Microbial Ecology	*strong policy*	1
Italian Journal of Zoology	*no policy*	1
Journal of Arid Environments	*no policy*	1
Journal of Evolutionary Biology	*JDAP membership*	9
Journal of Heredity†	*strong policy*	1
Journal of Mammalogy	*weak policy*	1
Journal of Mammalian Evolution	*no policy*	1
Journal of Theoretical Biology	*no policy*	1
Methods in Ecology and Evolution	*strong policy*	1
Molecular Biology and Evolution†	*weak policy*	2
Molecular Ecology	*JDAP membership*	8
Molecular Phylogenetics and Evolution	*no policy*	31
Nature†	*weak policy*	2
New Phytologist	*weak policy*	1
PLOS ONE†	*weak policy*	12
PLOS Biology†	*weak policy*	2
PeerJ	NA	1
Plant Systematics and Evolution	*weak policy*	2
PNAS	*weak policy*	11
Perspectives in Plant Ecology, Evolution and Systematics	*no policy*	1
Proceedings of the Royal Society B	*strong policy*	14
Science	*strong policy*	5
Systematic Entomology	*no policy*	2
Systematic Biology	*JDAP membership*	13
Trends in Microbiology	*no policy*	1
Virus Research	*no policy*	1

†Data-sharing policy in place at the time of study publication; has since become *JDAP membership.*
